# The Influence of Weakly Coordinating Cations on the O−H⋅⋅⋅O^−^ Hydrogen Bond of Silanol‐Silanolate Anions

**DOI:** 10.1002/chem.202004236

**Published:** 2020-12-07

**Authors:** Robin F. Weitkamp, Beate Neumann, Hans‐Georg Stammler, Berthold Hoge

**Affiliations:** ^1^ Centrum für Molekulare Materialien Fakultät für Chemie Universität Bielefeld Universitätsstraße 25 33615 Bielefeld Germany

**Keywords:** hydrogen bond, phosphazene bases, silanolates, silanol-silanolate, weakly coordinating cations

## Abstract

The reaction of a saline phosphazenium hydroxide hydrate with siloxanes led to a novel kind of silanol‐silanolate anions. The weakly coordinating behavior of the cation renders the formation of silanol‐silanolate hydrogen bonds possible, which otherwise suffer from detrimental silanolate–oxygen cation interactions. We investigated the influence of various weakly coordinating cations on silanol‐silanolate motifs, particularly with regard to different cation sizes. While large cations favor the formation of intramolecular hydrogen bonds resulting in cyclic structures, the less bulky tetramethyl ammonium cation encourages the formation of polyanionic silanol‐silanolate chains in the solid state.

## Graphical abstract

**Silanol‐silanolate anions**: The reactions of hydroxide salts featuring weakly coordinating cations with cyclic dimethylsiloxanes were investigated and afforded salts of the corresponding silanol‐silanolate anions. The formed hydrogen bonds are strongly affected by the size and coordination ability of the cation. While cyclic [D_3_OH]^−^ anions evolve in the presence of phosphazenium cations, linear polyanionic silanol‐silanolate strands are formed with [NMe_4_]^+^.

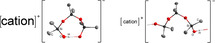

There is no life without hydrogen bonds.[^1^, ^2^, ^3^, ^4^, ^5^] The most prominent representative of hydrogen bonding is water. Without hydrogen bonding water would not exist in the well‐known form, but would exhibit significant differences in the melting and boiling point, as it is observed for the higher homologue H_2_S.^[6]^ Moreover, hydrogen bonding is crucial in supramolecular chemistry, as the helical structure of the DNA, as well as the secondary and tertiary structures of proteins and peptides, are based on those weak interactions.[^1^, ^2^, ^3^, ^4^, ^5^] Consequently, the understanding of hydrogen bonding is decisive to understand chemistry at all.

Hydrogen bonding is not an exclusive domain in biochemistry. Inorganic compounds besides H_2_O show such interactions as well. Silanols[^7^, ^8^, ^9^] and silanediols[^10^, ^11^, ^12^, ^13^] exhibit distinct tendencies for hydrogen bond formation, which often result in the formation of ring structures. Meanwhile several studies are directed to interactions in silanol‐silanol adducts,[^8^, ^9^, ^12^, ^13^, ^14^] as well as in silanol‐alcohol^[15]^ and in silanol‐amine[^9^, ^16^, ^17^] aggregates. The obtained structures are strongly affected by the presence of solvent molecules capable of hydrogen bonding. Silanols have also found application as catalysts in silanol‐hydrogen‐bond‐assisted coupling reactions[^10^, ^11^] and CO_2_ fixation strategies.[^16^, ^18^]

Weakly coordinating cations are essential for the investigation of hydrogen bonds in silanol‐silanolates, which is clearly clarified by the strong potassium oxygen interaction in the representative potassium silanolate [K{O(Ph_2_SiO)_2_SiPh_2_OH}]_2_ of Sullivan et al.^[19]^ The interaction of the silanolate oxygen with the potassium cation is favored over the formation of a silanol‐silanolate hydrogen bond. Thus, ring formation including the potassium ion is observed.

Bulky phosphazenium cations are predestinated for the investigation of non‐coordinated anions, such as naked fluoride anions,^[20]^ the hydroxide trihydrate anion^[21]^ or reactive aluminates,^[22]^ and are also capable to stabilize isolated silanol‐silanolate anions, as recently reported by us (Figure 1).^[23]^

**Figure 1:**
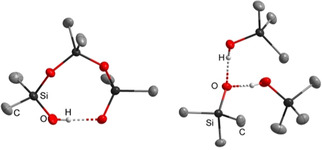
Depiction of selected molecular geometries of silanol‐silanolate anions in the solid state featuring the phosphazenium counterion **[1H]^+^**, reported earlier by us.^[23]^ The cation is not depicted.

The benefits of strong neutral bases like tetraphosphazene **1** ([(Et_2_N)_3_P=N]_3_P=N*t*Bu) and guanidino monophosphazene **2** ([(Me_2_N)_2_C=N]_3_P=N*t*Bu) applied in this work (Figure 2) are the high proton affinity paired with the low electrophilicity of the corresponding cations. In the following, we are going to show that in addition to the low coordination ability of these cations, their sizes also play decisive roles in the construction of the observed structural patterns.

**Figure 2:**
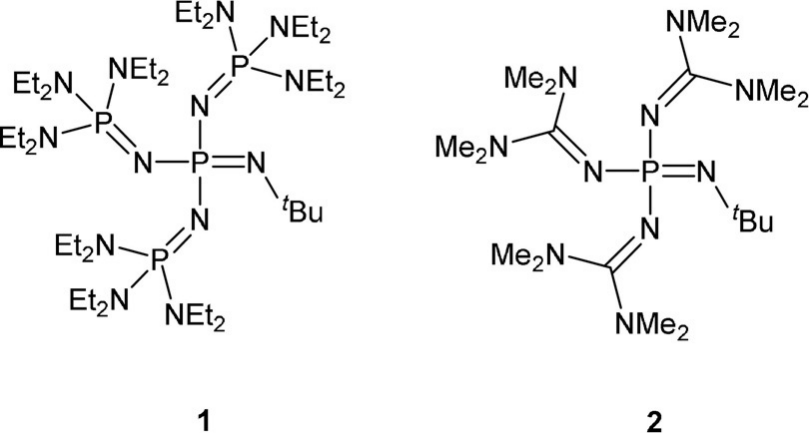
Phosphazene bases applied in this work.

As previously reported by us, silanol‐silanolate anions can be synthesized by the reaction of a saline phosphazenium hydroxide hydrate with siloxane species.[^23^, ^24^] Due to the weak electrophilicity of the cation, anions are formed in which hydrogen bond formation is favored over cation anion interactions. We disclosed that hydrogen bonding is crucial for the existence of highly basic silanolate anions. Similar to the decomposition pathway of the hydroxide trihydrate anion, non‐coordinated silanolate anions are not viable in the presence of phosphazenium cation **[1H]^+^** due to deprotonation of the cation.^[23]^ The selective syntheses of silanol‐silanolate anions without cation anion interactions render a precise investigation of hydrogen bond formation possible. In the case of dimethyl‐ (Figure 1, left) or diphenyl‐siloxanes^[23]^ the corresponding cyclic [D_3_OH]^−^ anions featuring strong intramolecular hydrogen bonds are particularly stable in the solid state. This is in agreement with the results of Baney and Atkari, who proposed the formation of [D_3_OH]^−^ anions by potentiometric titration experiments of cyclic siloxanes with tetra‐*n‐*butylammonium hydroxide.^[25]^ In order to examine cation‐dependent differences in silanol‐silanolate structures, hydroxide salts of the depicted cations in Scheme 1 were applied for the reaction with dimethylsiloxanes.

**Scheme 1:**
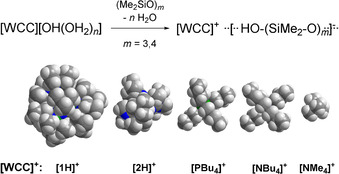
Reaction of hydroxide hydrate salts of weakly coordinating cations (WCC) with cyclic siloxanes (top) and space filling models of weakly coordinating cations obtained from X‐ray investigations (below).

The free tetra‐ and monophosphazene bases **1** ^[21]^ and **2**[^24^, ^27^] were synthesized according to known literature procedures. The latter was liberated from its hydrochloride salt applying NaNH_2_ in liquid ammonia as the deprotonation agent.

**[1H][OH(OH_2_)**_***n***_ **]** was generated by addition of one molar equivalent of water to the free base in *n*‐hexane. The subsequent reaction with hexamethylcyclotrisiloxane (D_3_) afforded **[1H][D_3_OH]** in a nearly quantitative yield on a multigram scale. The non‐hygroscopic product is well soluble in benzene and chlorobenzene. The ^29^Si NMR resonance of the silicon atoms adjacent to the intramolecular hydrogen bridge appears as a singlet at *δ*=−23.9 ppm. The signal of the central silicon atom is slightly highfield shifted to *δ*=−24.1 ppm. The intramolecular hydrogen bond length (O1–O4 distance) amounts to 242.8(2) pm (Figure 1).[^23^, ^26^]

Whereas the cation of salt **[1H][OH(OH_2_)**_***n***_ **]** is highly resistant towards hydroxide anions in THF or aqueous solution, phosphazene **2** hydrolyses in H_2_O via its hydroxide salt. ^31^P NMR resonances of unspecified products at *δ*=−5.4, −0.2 and 1.7 ppm were observed. The resonance at *δ*=19.7 ppm is assigned to the phosphorus atom of phosphane oxide [(Me_2_N)_2_C=N]_3_P=O. In the presence of NaOMe in aqueous MeOH, hydrolysis of base **2** leads to the formation of the amide derivative **3**, with a ^31^P NMR resonance at *δ*=−4.8 ppm in water. The product was further evidenced by X‐ray diffraction (Scheme 2).^[26]^

**Scheme 2:**
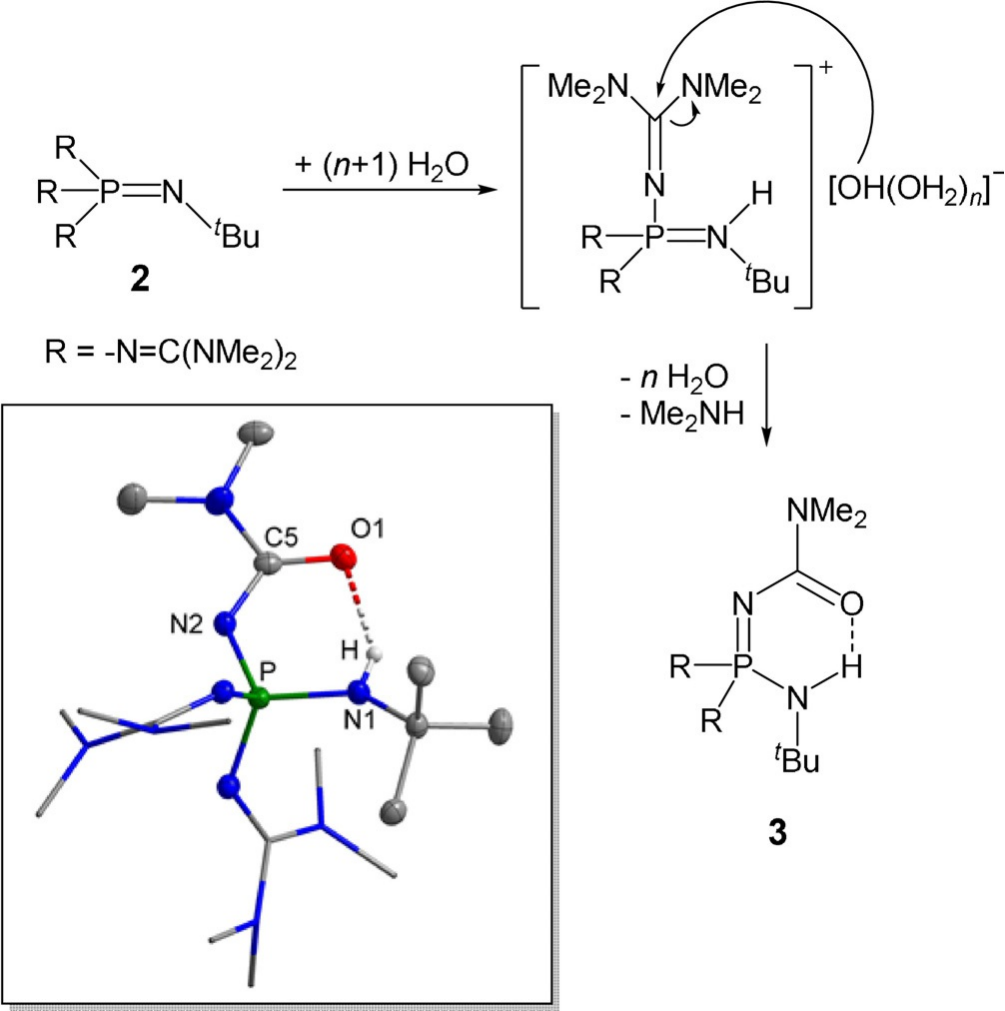
Hydrolysis of **2** and molecular structure of **3** (box): All hydrogen atoms bonded at carbon atoms are omitted for clarity.^[26]^ Guanidyl groups are shown as stick model. Selected bond lengths [pm]: O1−C5 125.2(5), N2−C5 134.3(5).

The amide **3** is characterized by a cyclic intramolecular hydrogen bridge. The corresponding O1–N1 distance amounts to 278.9(4) pm.

Hydrolysis of **[2H]^+^** can be efficiently suppressed temporarily if siloxanes like D_3_ are present in the mixture prior to the addition of water (Scheme 3).

**Scheme 3:**
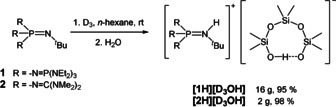
Syntheses of phosphazenium silanol‐silanolates.

Thus, the corresponding guanidino phosphazenium salt **[2H][D_3_OH]** was isolated as pale yellow, highly hygroscopic crystals (98 % yield). However, in the presence of [D_3_OH]^−^ cation **[2H]^+^** succumbs quite fast to hydrolysis at ambient temperature also resulting in the clean formation of **3**, which hampers a reliable elemental analysis. In the ^31^P NMR spectrum of **[2H][D_3_OH]** the resonance of the phosphorus atom in **[2H]^+^** is observed as a singlet at *δ*=−10.6 ppm. In the ^1^H^29^Si HMBC NMR spectrum the resonances of the terminal silicon atoms of the anionic ring are slightly downfield shifted (*δ*=−22.8 ppm) compared to **[1H][D_3_OH] (** *δ*=−23.7 ppm).

An X‐ray diffraction study of crystals grown from the cooled reaction mixture reveals a cation‐anion hydrogen bond interaction with an N1‐O4 separation of 285.2(2) pm (Figure 3), which is presumably responsible for the small downfield shift of the terminal ^29^Si nuclei.^[26]^ The other hydrogen bond within the anion with an O1–O4 distance of 250.8(2) pm is elongated compared to **[1H][D_3_OH]** (242.8(2) pm).

**Figure 3:**
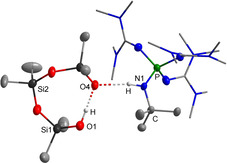
Molecular structure of **[2H][D_3_OH]** with highlighted cation anion hydrogen bond interaction.^[26]^ Disorder of Si1, C22 and C23 over two sites (76:24). Disordered parts and hydrogen atoms of methyl groups are omitted for clarity. Guanidyl groups are shown as stick model. Selected bond lengths [pm] and angles [°]: N1−P1 162.9(1), N1−O4 285.2(2), O1−O4 250.8(2), O1−Si1 160.7(1), O4−Si3 159.0(1); O1‐O4‐Si3 116.2(1), O4‐O1‐Si1 113.9(1), O2‐Si2‐O3 111.9(3).

The salt **[2H][D_3_OH]** decomposes within days at room temperature, and it deteriorates fast above 75 °C with hydrolysis of its cation under formation of **3** (^31^P shift of the decomposition product at *δ*=1.0 ppm in chlorobenzene, lock with [D_6_]acetone in a capillary) and liberation of cyclic siloxanes, mainly D_4_.

For the syntheses of phosphonium and ammonium silanolates, the corresponding hydroxides ([NMe_4_]OH 25 wt. % in MeOH, [NBu_4_][OH(OH_2_)_30_], [PBu_4_]OH 40 wt. % in H_2_O) were applied. The nature of the employed siloxane source is not decisive, since equilibrium mixtures of linear and cyclic siloxanes are always present in the base assisted reaction and D_3_ fragments are formed by binding rearrangements. We employed liquid octamethylcyclotetrasiloxane (D_4_), which additionally acted as the solvent. In the case of [NMe_4_]OH, the reaction with D_3_ as siloxane source was performed in ethereal solution.^[24]^ In all cases the excess of siloxane and water can be removed in vacuo after the reaction.^[23]^ The tetra‐*n*‐butylammonium and tetra‐*n*‐butylphosphonium silanolates were isolated in yields of 92 % and 73 % after crystallization from saturated ethereal solutions at −28 °C. The ^29^Si NMR spectrum of **[NBu_4_][D_3_OH]** is depicted in Figure 4. The resonance at *δ*=−24.4 ppm results from the silicon atoms adjacent to the hydrogen bond.

**Figure 4:**
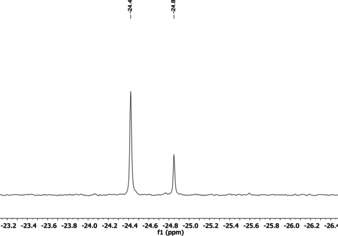
^29^Si NMR spectrum of **[NBu_4_][D_3_OH]** in Et_2_O (lock with [D_6_]acetone in a capillary).

The X‐ray structural investigation reveals the cyclic [D_3_OH]^−^ anion in both salts, which is depicted for **[NBu_4_][D_3_OH]** as a representative example in Figure 5.^[26]^

**Figure 5:**
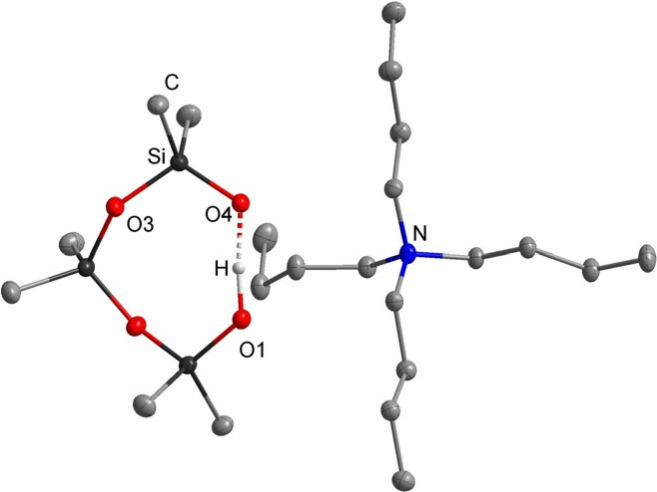
Molecular structure of **[NBu_4_][D_3_OH]**.^[26]^ All hydrogen atoms bonded at carbon atoms are omitted for clarity. Thermal ellipsoids are set at 50 % probability. Selected bond length [pm]: O1‐O4 245.1(1).

The hydrogen bonds within the obtained [D_3_OH]^−^ anions were identified by the O1‐O4 distances, which amount to 245.1(1) pm for **[NBu_4_][D_3_OH]** and 244.3(1) pm for **[PBu_4_][D_3_OH]**. They are well comparable with the value in **[1H][D_3_OH]**.^[23]^ Both salts are highly hygroscopic and melt within seconds under air. The salt **[NBu_4_][D_3_OH]** completely decomposes by a Hofmann‐type elimination reaction of the cation above 80 °C in high vacuum. The released products tributylamine and butene were detected via ^1^H NMR spectroscopy. Cyclic siloxanes are also liberated.^[24]^ While **[NBu_4_][D_3_OH]** is stable at room temperature as a crystalline solid under inert atmosphere over months without any traces of decomposition, the tetra‐*n‐*butylphosphonium cation in solid **[PBu_4_][D_3_OH]** completely hydrolyzes within days under the same conditions. Fast hydrolysis is observed over a few hours in ethereal solution to afford the corresponding tributylphosphane oxide, which is evidenced by ^31^P NMR spectroscopy at a chemical shift of *δ*=42.5 ppm (lit: 42.0 ppm).^[28]^

From the corresponding reaction of [NMe_4_]OH with D_3_ a colorless sticky oil was obtained, which is insoluble in benzene and barely soluble in diethyl ether and chlorobenzene. By layering the oil with diethyl ether and storage at −28 °C, colorless crystals of **[NMe_4_][D_3_OH]_1/∞_** were obtained with a melting point of 75 °C. Its decomposition at ambient temperature, which impedes a reliable elemental analysis, is accompanied by a strong amine odor and a yellow discoloration. The ^29^Si NMR spectroscopic investigation of a milky chlorobenzene solution of the mixture reveals the presence of six chemically inequivalent silicon atoms. In comparison to previously discussed [WCC][D_3_OH] salts, the resonances at *δ*=−24.7 and −24.1 ppm in a ratio of 2:1 are assigned to the silicon atoms of the [D_3_OH]^−^ moiety. The X‐ray diffraction of a small crystalline fragment reveals a polymeric silanolate anion in **[NMe_4_][D_3_OH]_1/∞_** (Figure 6).^[26]^ In stark contrast to cyclic silanol‐silanolate anions of the type [D_3_OH]^−^, which were obtained with bulky counterions, the small tetramethylammonium cation favors the formation of intermolecular hydrogen bonding in the solid state. Compared to the cyclic anion in **[NBu_4_][D_3_OH]**, in which the terminal silanol and silanolate functions exhibit torsion angles of 55.9° (O1‐Si1‐O2‐Si2) and 55.8° (O4‐Si3‐O3‐Si2), the terminal functions in **[NMe_4_][D_3_OH]_1/∞_** are rotated around the bonds Si1‐O2 and Si3‐O3 with torsion angles of 123.9° (O1‐Si1‐O2‐Si2) and 132.5° (O4‐Si3‐O3‐Si2). This gives rise to linear polyanionic chains of [D_3_OH]_1/∞_^−^ units (Figure 6), which seem to be responsible for the poor solubility of salt **[NMe_4_][D_3_OH]_1/∞_**.

**Figure 6:**
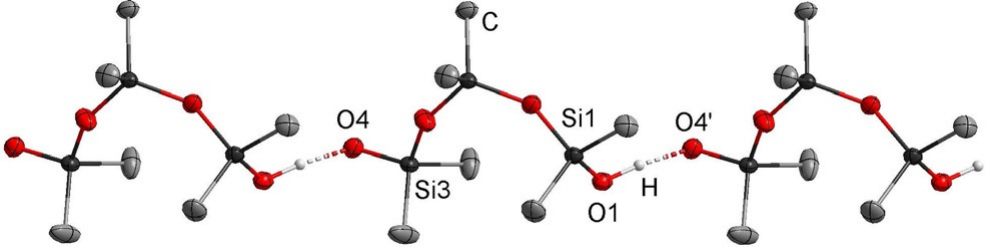
Section of the strand of the [D_3_OH]^−^_*x*_ polyanion along the *x* axis spanned by intermolecular silanol‐silanolate hydrogen bonding in **[NMe_4_][D_3_OH]_1/∞_**.^[26]^ All hydrogen atoms bonded at carbon atoms and tetramethylammonium counterions are omitted for clarity. Thermal ellipsoids are set at 50 % probability. Selected bond length [pm]: O1−O4’ 247.0(1).

The intermolecular O1–O4’ distance was determined to 247.0(1) pm and is thus slightly elongated in comparison to intramolecular hydrogen bonding in **[1H][D_3_OH]** (242.8(2) pm) and **[NBu_4_][D_3_OH]** (245.1(1) pm). Interestingly, the anionic structural motif of the three SiMe_2_O units is maintained.

Accidentally, impurities of potassium hydroxide were present in a flask of [NMe_4_]OH and cyclic siloxanes. Gratifyingly, single crystals of salt **[NMe_4_][K(D_3_OH)_2_]** evolved and were analyzed by X‐ray crystallography (Figure 7).^[26]^

**Figure 7:**
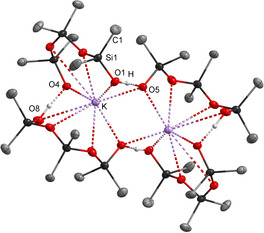
Molecular structure of the dimer of **[NMe_4_][K(D_3_OH)_2_]**.^[26]^ The ammonium cations are not shown. All hydrogen atoms linked to carbon atoms are omitted for clarity. Thermal ellipsoids are set at 50 % probability. Selected bond lengths [pm]: K−O4 269.1(2), O1−O5 244.5(2), O4−O8’ 249.6(2).

The potassium cation is surrounded by two [D_3_OH]^−^ units exhibiting strong potassium silanolate (*d*(K–O4)=269.1(2) pm) and silanol‐silanolate interactions (*d*(O4–O8’)=249.6(2) pm) in a mono‐anionic complex. Moreover, this complex is associated to a dimer in the solid state, displaying strong potassium silanolate interactions with a K–O5 separation of 274.4(1) ppm and additional silanol‐silanolate hydrogen bonds (*d*(O1–O5)=244.5(2) pm). Again, the structural motif of the three SiMe_2_O units remains favorable. Similar to the potassium salt presented by Sullivan et al. previously,^[19]^ the potassium ions form strong interactions to silanolate oxygen atoms, which obviously significantly influenced the construction of the formed pattern. This example impressively underlines the need for weakly coordinating cations to observe isolated silanol‐silanolate interactions.

In conclusion we succeeded in the clean formation of silanol‐silanolate salts featuring weakly coordinating cations. In all cases [D_3_OH]^−^ anions containing three siloxane units were delivered, which proves the extraordinary thermal stability of this motif. Phosphazenium silanol‐silanolate salts with cyclic anions of the type [D_3_OH]^−^ in **[1H][D_3_OH]** and **[2H][D_3_OH]** were obtained in excellent yields (>95 %) by the reaction of the free phosphazene bases with water and cyclodimethylsiloxanes. The corresponding tetra‐*n*‐butylammonium and ‐phosphonium salts **[NBu_4_][D_3_OH]** and **[PBu_4_][D_3_OH]** were afforded analogously by use of their hydroxides. All cyclic [D_3_OH]^−^ anions show intramolecular hydrogen bonding, with O–O distances depending on the size and the coordination capability of the applied cation. While an enlargement of non‐coordinating cations from [NBu_4_]^+^ to **[1H]^+^** results in a shortening of the intramolecular hydrogen bond from 245.1(1) pm to 242.8(2) pm, hydrogen bond donating cations like **[2H]^+^** favor an extension of the formed bond (250.8(2) pm). Downsizing the cation to [NMe_4_]^+^ in **[NMe_4_][D_3_OH]_1/∞_** benefits the organization of the [D_3_OH]^−^ moieties in a polyanionic strand. In contrast to cyclic [D_3_OH]^−^ anions, an intermolecular hydrogen bond (247.0(1) pm) is predominant in the presence of less bulky counterions.

## Conflict of interest

The authors declare no conflict of interest.

## Supporting information

As a service to our authors and readers, this journal provides supporting information supplied by the authors. Such materials are peer reviewed and may be re‐organized for online delivery, but are not copy‐edited or typeset. Technical support issues arising from supporting information (other than missing files) should be addressed to the authors.

Supplementary
