# Supplementary material for: The Influence of Weakly Coordinating Cations on the O−H⋅⋅⋅O− Hydrogen Bond of Silanol‐Silanolate Anions
Source: Chemistry. 2020 Dec 7;27(3):915–20. doi: 10.1002/chem.202004236 (PMC7839788; doi:10.1002/chem.202004236)
Supplement: Supplementary file 1 — Supplementary [file CHEM-27-915-s001.pdf]

Chemistry–A European Journal

Supporting Information

# The Influence of Weakly Coordinating Cations on the O–H···O$^-$ Hydrogen Bond of Silanol-Silanolate Anions

Robin F. Weitkamp, Beate Neumann, Hans-Georg Stammler, and Berthold Hoge*[a]

# 1. Experimental Section

## 1.1 General Part

All chemicals were obtained from commercial sources and used without further purification. Standard high-vacuum techniques were employed throughout all experiments. Non-volatile compounds were handled in a dry $N_2$ atmosphere using Schlenk techniques.

## 1.2 Analysis Methods

### 1.2.1 NMR Spectroscopy

NMR spectra were recorded on a Bruker Model Avance III 300 spectrometer ($^{1}H$ 300.13 MHz; $^{13}C$ 75.47 MHz; $^{29}Si$ 59.63 MHz; $^{31}P$ 121.49 MHz) or on a Bruker Avance III 500 spectrometer ($^{1}H$ 500.01 MHz; $^{13}C$ 125.73 MHz; $^{29}Si$ 99.34 MHz; $^{31}P$ 202.41 MHz) or on a Bruker Avance III 500 HD spectrometer ($^{1}H$ 500.20 MHz; $^{13}C$ 125.79 MHz; $^{29}Si$ 99.38 MHz; $^{31}P$ 202.48 MHz). Positive shifts are downfield from the external standards TMS ($^{1}H$, $^{13}C$, $^{29}Si$) and $H_3PO_4$ ($^{31}P$). The NMR spectra were recorded in the indicated deuterated solvent or in relation to acetone-$d_6$ filled capillaries.

### 1.2.2 IR Spectroscopy

IR spectra were recorded on an ALPHA-FT-IR spectrometer (Bruker) using an ATR unit with a diamond crystal for liquids and solids.

### 1.2.3 Elemental Analyses

Elemental analyses were performed by Mikroanalytisches Laboratorium Kolbe (Oberhausen, Germany).

### 1.2.4 Melting Point

Melting points were measured on a Mettler Toledo Mp70 Melting Point System.

## 1.3 Syntheses

### 1.3.1 Synthesis of [1H][$D_3$OH]

Phosphazene **1** (13.69 g, 15.5 mmol) is dissolved in 45 mL of *n*-hexane before hexamethylcyclotrisiloxane (3.44 g, 15.5 mmol) is added. After addition of water (0.29 g, 15.5 mmol) two phases separated. After stirring at room temperature overnight, a pale yellow solid precipitates. The supernatant solution is removed via a syringe and the solid is dried in a high vacuum ($10^{-3}$ mbar). The product (16.46 g, 14.6 mmol, 95 % based on **1**) is obtained as a colorless crystalline solid (m.p. 99-101 °C).

$^{1}$H NMR ($C_6D_6$, rt): $\delta$ [ppm] = 0.6 (s, 6 H, SiO($H_3C$)$_2$SiOSi), 0.7 (s, 12 H, ($H_3C$)$_2$SiOH-OSi($CH_3$)$_2$), 1.0 (t, $^{3}J_{HH}$ = 7 Hz, 54 H, $CH_3$), 1.3 (s, 9 H, C($CH_3$)$_3$), 2.1 (d, $^{2}J_{PH}$ = 8 Hz, 1 H, NH), 3.0 (d, q, $^{3}J_{PH}$ = 10 Hz, $^{3}J_{HH}$ = 7 Hz, 36 H, $CH_2$), 14.0 (s, OH).
$^{13}$C{$^{1}$H} NMR ($C_6D_6$, rt): $\delta$ [ppm] = 2.3 (s, SiO($H_3C$)$_2$SiOSi), 3.6 (s, ($H_3C$)$_2$SiOH-OSi($CH_3$)$_2$), 13.4 (d, $^{3}J_{PC}$ = 4 Hz, $CH_3$), 31.3 (d, $^{3}J_{PC}$ = 5 Hz, C(**C**$H_3$)$_3$), 39.0 (d, $^{2}J_{PC}$ = 6 Hz, $CH_2$), 50.4 (d, $^{2}J_{PC}$ = 4 Hz, **C**($CH_3$)$_3$).
$^{29}$Si{$^{1}$H}dept30 NMR ($C_6D_6$, rt): $\delta$ [ppm] = -24.1 (s, SiO($H_3C$)$_2$**Si**OSi), -23.9 (s, ($H_3C$)$_2$**Si**OH-O**Si**($CH_3$)$_2$).
$^{29}$Si{$^{1}$H}IG NMR (PhCl, rt): $\delta$ [ppm] = -24.2 (s, SiO($H_3C$)$_2$**Si**OSi), -23.7 (s, ($H_3C$)$_2$**Si**OH-O**Si**($CH_3$)$_2$).
$^{31}$P NMR ($C_6D_6$, rt): $\delta$ [ppm] = -33.7 (q, d, $^{2}J_{PP}$ = 70 Hz, $^{2}J_{PH}$ = 8 Hz, 1 P, P=NH), 7.6 (d, tridec, $^{2}J_{PP}$ = 70 Hz, $^{3}J_{PH}$ = 10 Hz, 3 P, ($Et_2N$)$_3$P).
IR (ATR): $\tilde{\nu}$ [cm$^{-1}$] = 2966 (vw), 2872 (vw), 1627 (vw, vbr), 1464 (vw), 1379 (w), 1351 (w), 1273 (m, br), 1247 (m), 1202 (m), 1175 (s), 1053 (w), 1016 (vs), 940 (vs), 847 (w), 784 (vs), 700 (m), 614 (w), 508 (m), 440 (m).
elemental analysis of $C_{46}H_{119}N_{13}O_4P_4Si_3$ (M = 1126.7 g/mol): calcd.: C 49.04, H 10.65, N 16.16, P 11.00, Si 7.48; found: C 48.61, H 10.64, N 15.89, P 10.98, Si 7.59.

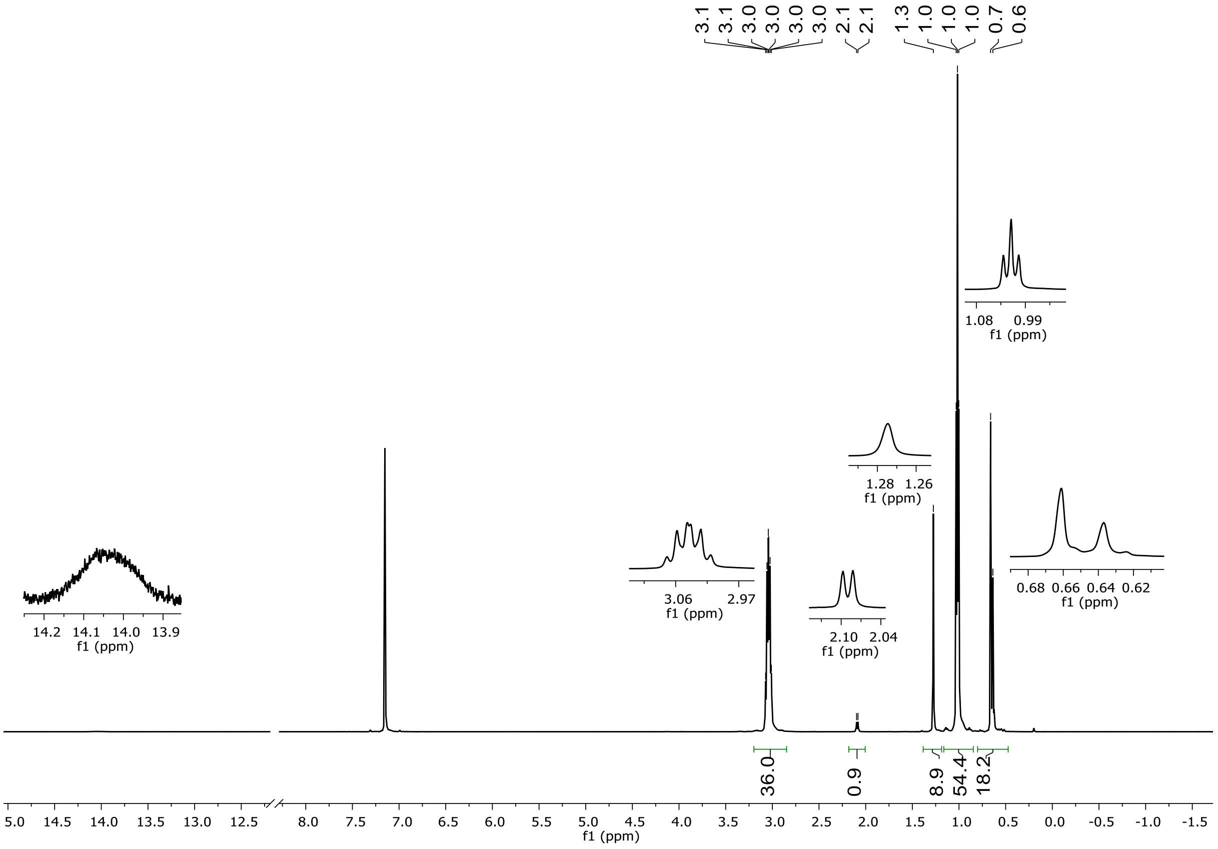


**Figure 1.** $^{1}H$ NMR spectrum of **[1H][D$_3$OH]** in benzene-d$_6$ (500 MHz).

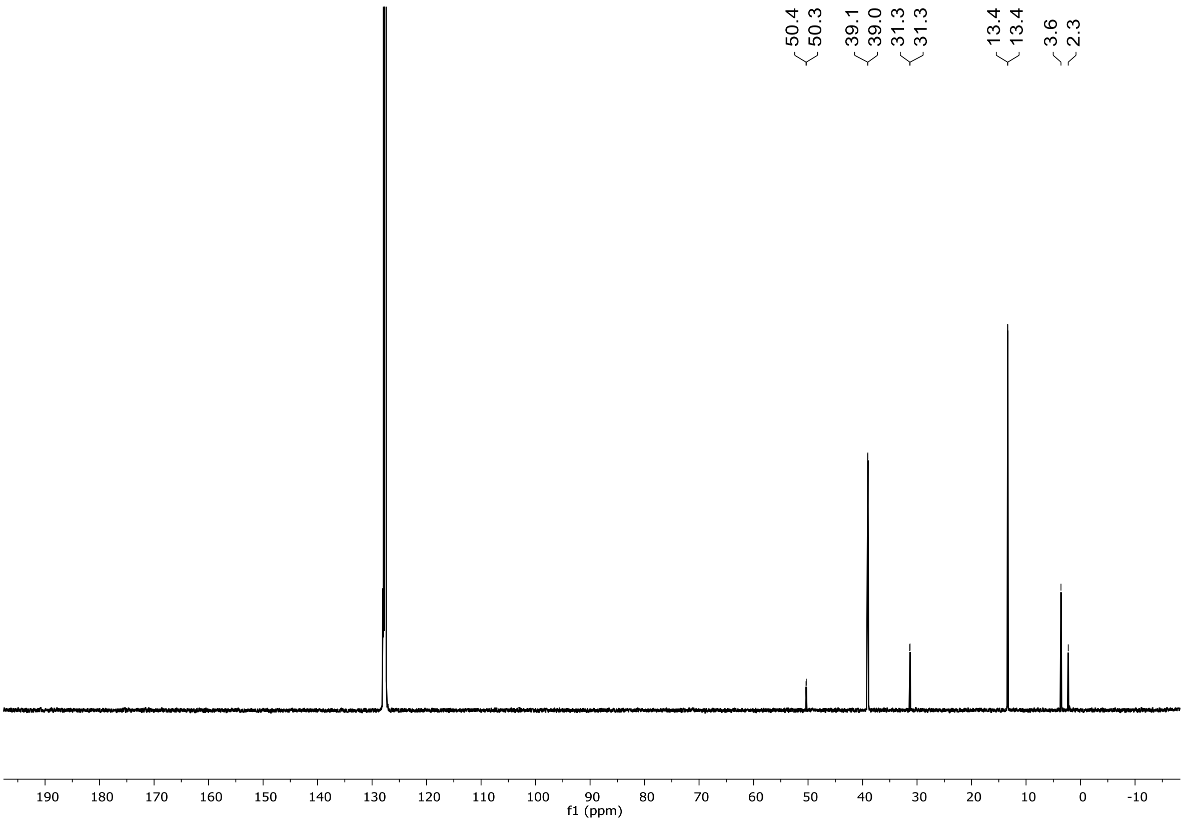


**Figure 2.** $^{13}C\{^{1}H\}$ NMR spectrum of **[1H][D$_3$OH]** in benzene-d$_6$ (500 MHz).

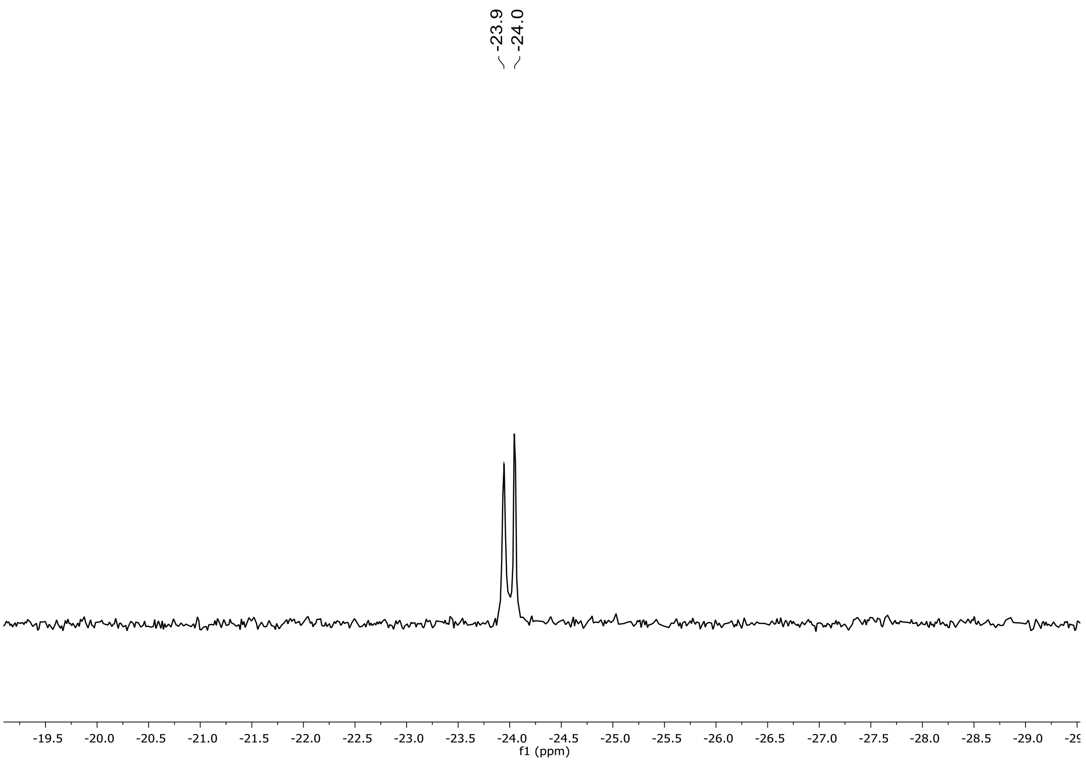


**Figure 3.** $^{29}Si\{^{1}H\}$dept30 NMR spectrum of **[1H][D$_3$OH]** in benzene-d$_6$ (500 MHz).

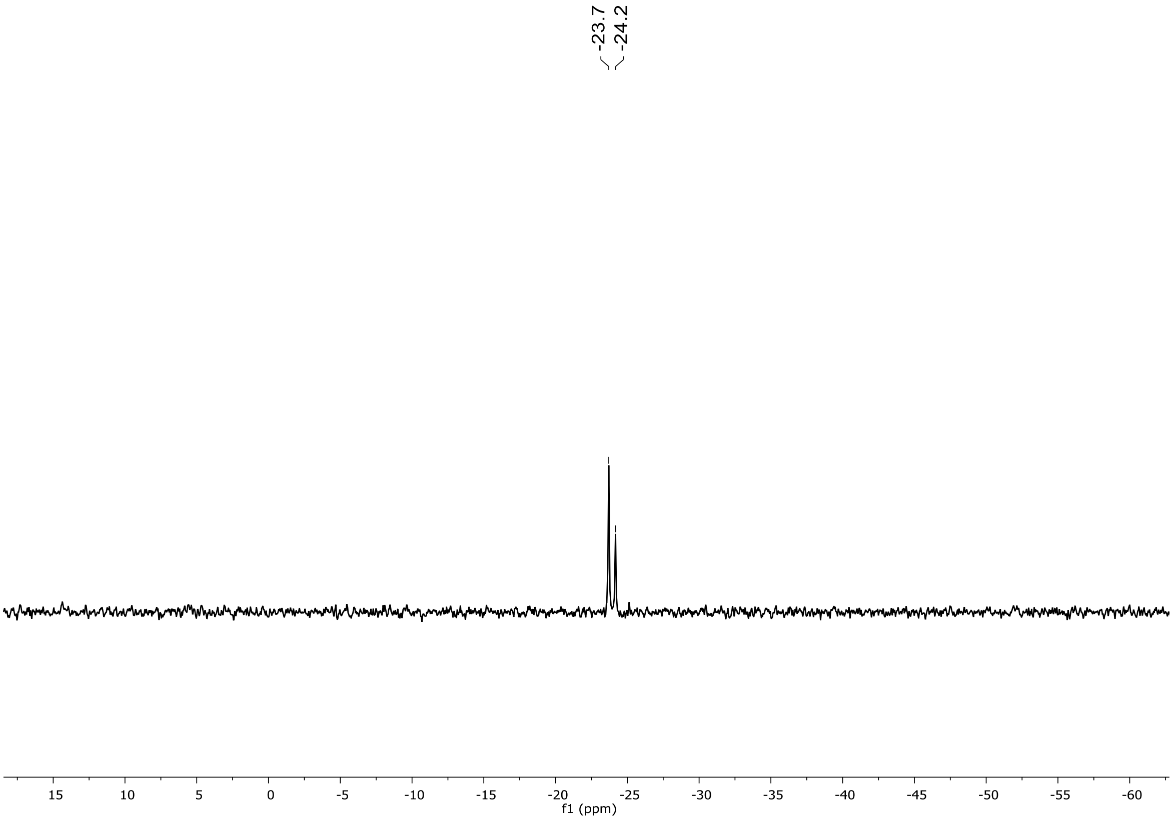


**Figure 4.** $^{29}Si\{^{1}H\}$IG NMR spectrum of **[1H][D$_3$OH]** in PhCl (300 MHz). Lock with acetone-d$_6$ in a capillary.

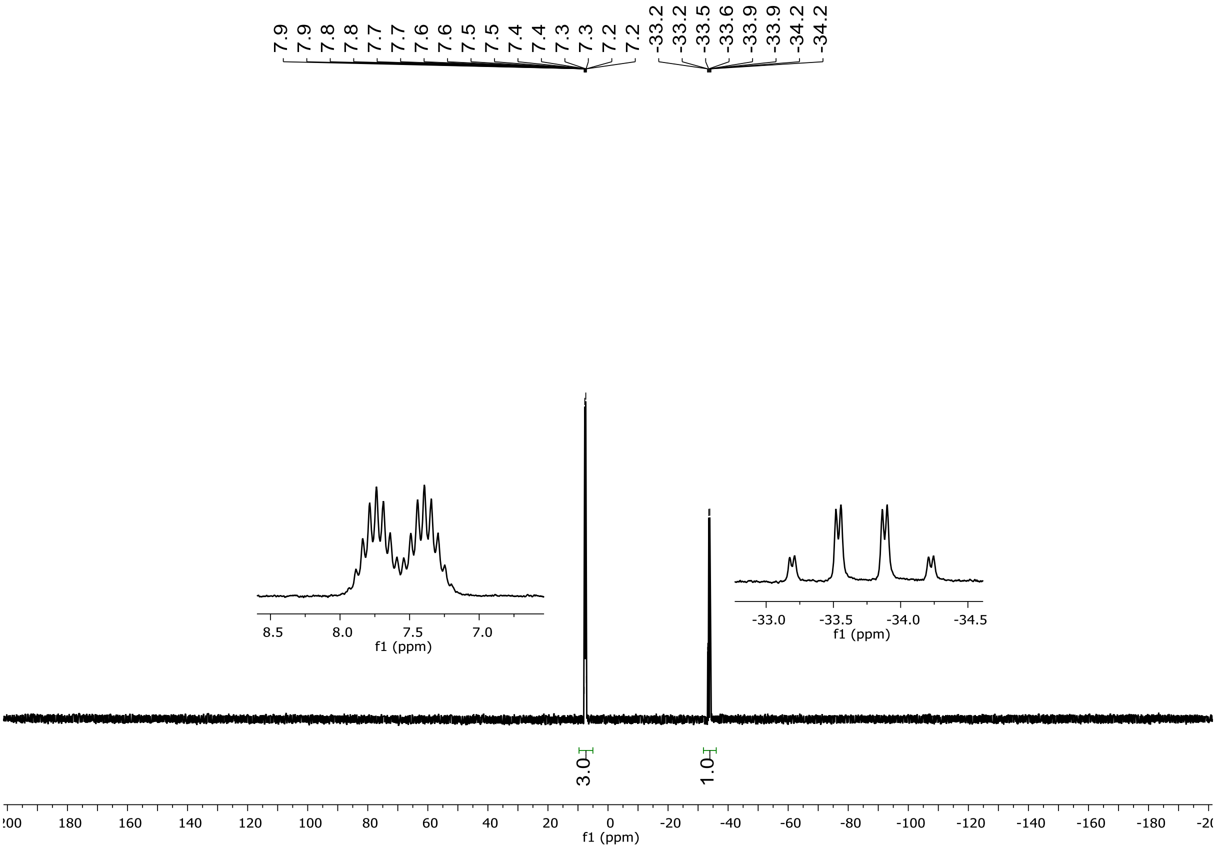


**Figure 5.** $^{31}$P NMR spectrum of **[1H][D$_3$OH]** in benzene-d$_6$ (500 MHz).

### 1.3.2 Synthesis of [2H][$D_3OH$]

To a solution of **2** (1.16 g, 2.61 mmol) in 10 mL of *n*-hexane first hexamethylcyclotrisiloxane (593 mg, 2.67 mmol) and then water (57 mg, 3.16 mmol) are added to yield a yellowish second phase. After one hour of stirring, the emulsion was kept at -28 °C overnight, by which a solid precipitated. The supernatant is removed via a syringe and the product (1.75 g, 2.55 mmol, 98 % based on **2**) is isolated as a pale yellow crystalline solid (m.p. 75 °C (dec.)).

The product decomposes at ambient temperature by hydrolysis of **[2H]$^+$**, which is accompanied by a strong amine odor. The $^{31}$P NMR resonance of the decomposition product **3** is observed as a singlet at δ = 2.2 ppm in benzene-$d_6$.

$^{1}$H NMR (PhCl, rt): *δ* [ppm] = 0.1 (m, 18 H, $(H_3C)_2SiO$), 1.2 (s, 9 H, $C(CH_3)_3$), 2.5 (s, 36 H, $N(CH_3)_2$), 9.7 (s, br, SiOH).

$^{13}$C{$^{1}$H}APT NMR (PhCl, rt): *δ* [ppm] = 1.9 (s, $SiO(H_3C)_2SiOSi$), 3.1 (s, $(H_3C)_2SiOH$-$OSi(CH_3)_2$), 31.2 (d, $^{3}J_{PC}$ = 4 Hz, $C(\mathbf{C}H_3)_3$), 39.8 (s, $N(CH_3)_2$), 50.0 (s, $\mathbf{C}(CH_3)_3$), 159.2 (s, C=N).

$^{1}$H$^{29}$Si HMBC NMR (PhCl, rt): *δ* [ppm] = 0.1 / -24.0 ($SiO(\mathbf{H}_3C)_2\mathbf{Si}OSi$), 0.1 / -22.8 (s, $(\mathbf{H}_3C)_2\mathbf{Si}OH$-$O\mathbf{Si}(C\mathbf{H}_3)_2$).

$^{29}$Si{$^{1}$H} IG NMR (PhCl, rt): *δ* [ppm] = -24.2 (s, $SiO(H_3C)_2\mathbf{Si}OSi$), -23.4 (s, $(H_3C)_2\mathbf{Si}OH$-$O\mathbf{Si}(CH_3)_2$).

$^{31}$P NMR (PhCl, rt): *δ* [ppm] = -10.6 (s, P=NH).

IR (ATR): $\tilde{\nu}$ [$cm^{-1}$] = 3008 (vw), 2951 (vw), 2933 (vw), 2896 (vw), 2813 (vw), 1617 (vw), 1543 (vs), 1504 (s), 1472 (m), 1421 (m), 1404 (m), 1375 (s), 1232 (m), 1135 (m), 1044 (s), 1010 (vs), 982 (m), 917 (s), 892 (vs), 787 (vs), 753 (s), 657 (s), 641 (m), 596 (m), 579 (w), 553 (m), 525 (w), 511 (w), 458 (w), 436 (m).

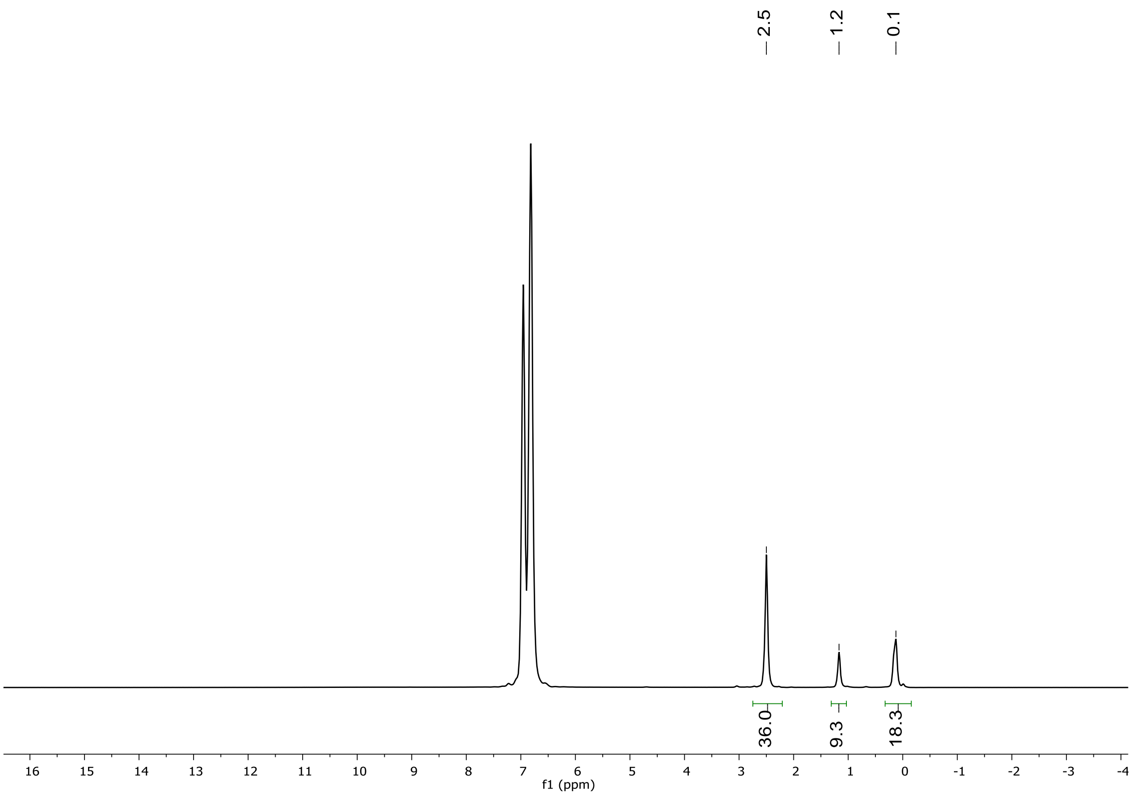


**Figure 6.** $^{1}H$ NMR spectrum of **[2H][D$_3$OH]** in PhCl. Lock with acetone-d$_6$ in a capillary (300 MHz).

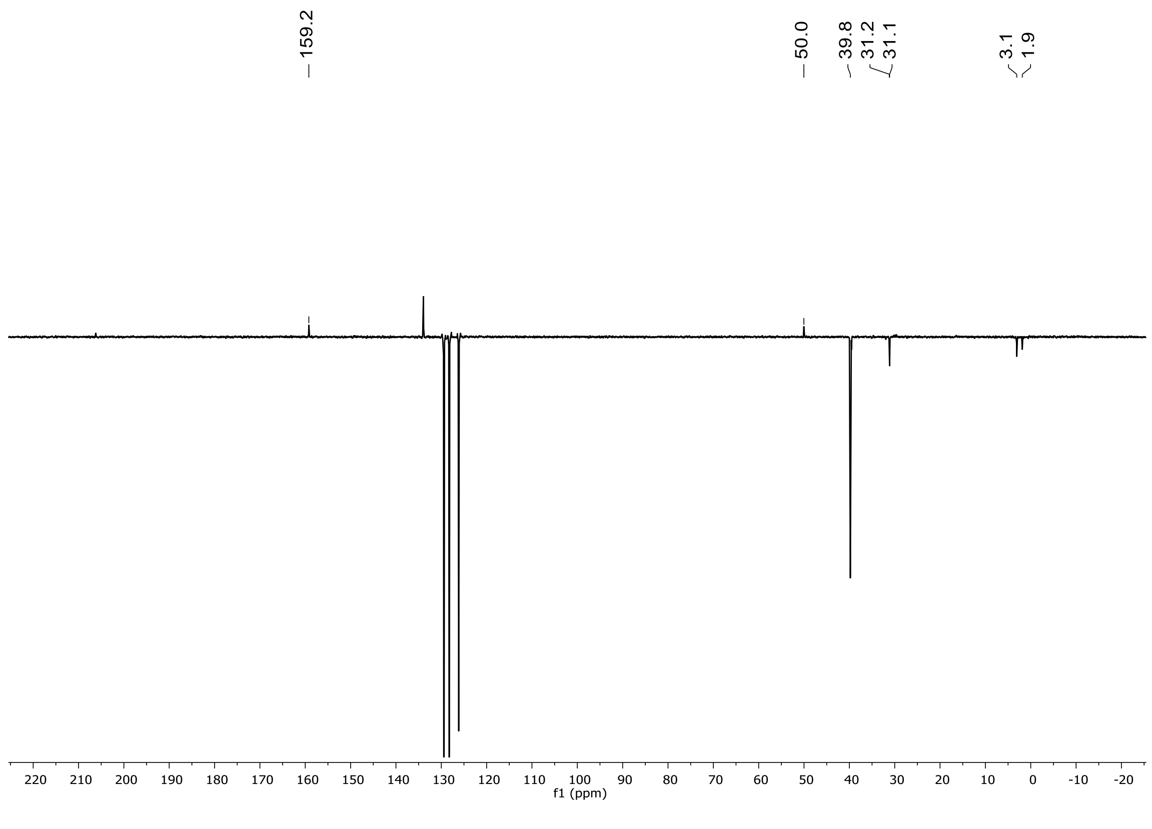


**Figure 7.** $^{13}C\{^{1}H\}$APT NMR spectrum of **[2H][D$_3$OH]** in PhCl. Lock with acetone-d$_6$ in a capillary (300 MHz).

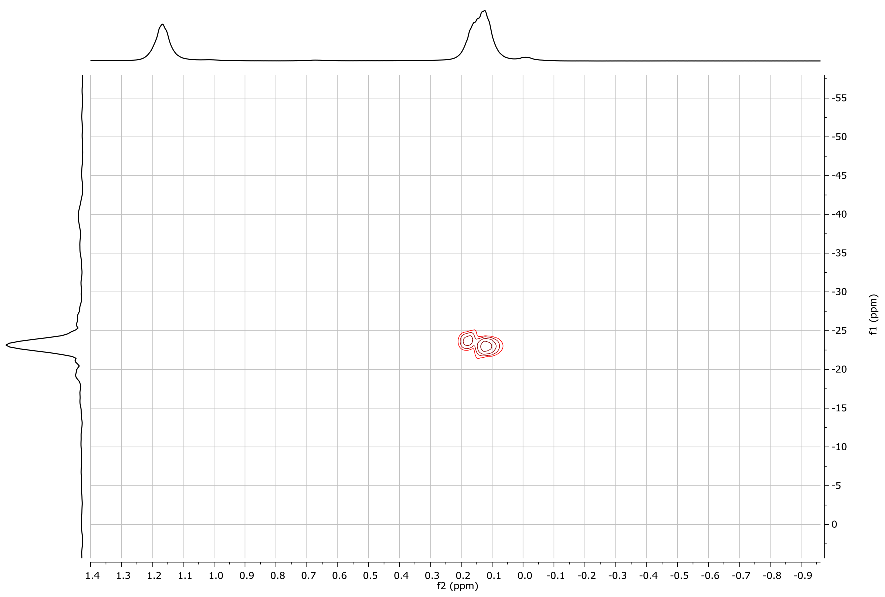


**Figure 8.** $^1H^{29}Si$ HMBC NMR spectrum of **[2H][D$_3$OH]** in PhCl. Lock with acetone-d$_6$ in a capillary (300 MHz).

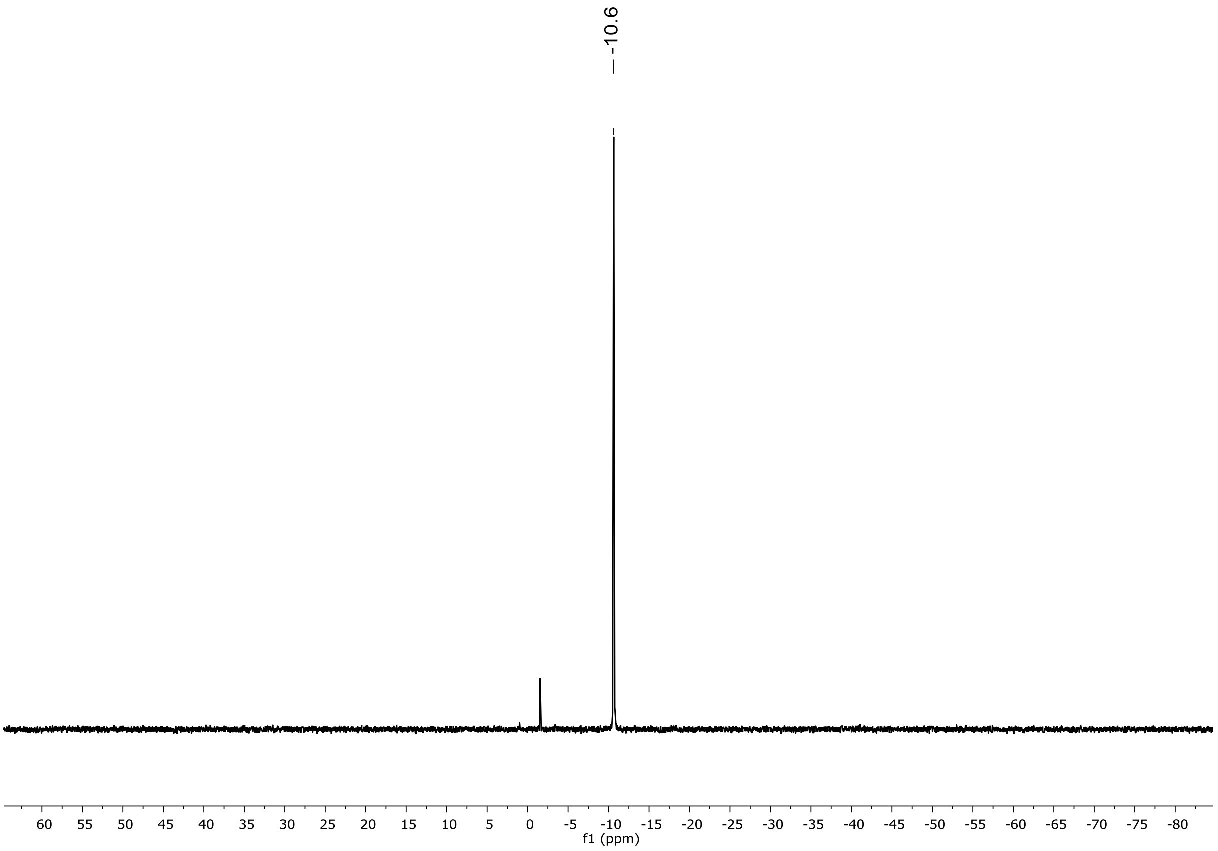


**Figure 9.** $^{31}P$ NMR spectrum of **[2H][D$_3$OH]** in PhCl. Lock with acetone-d$_6$ in a capillary (300 MHz).

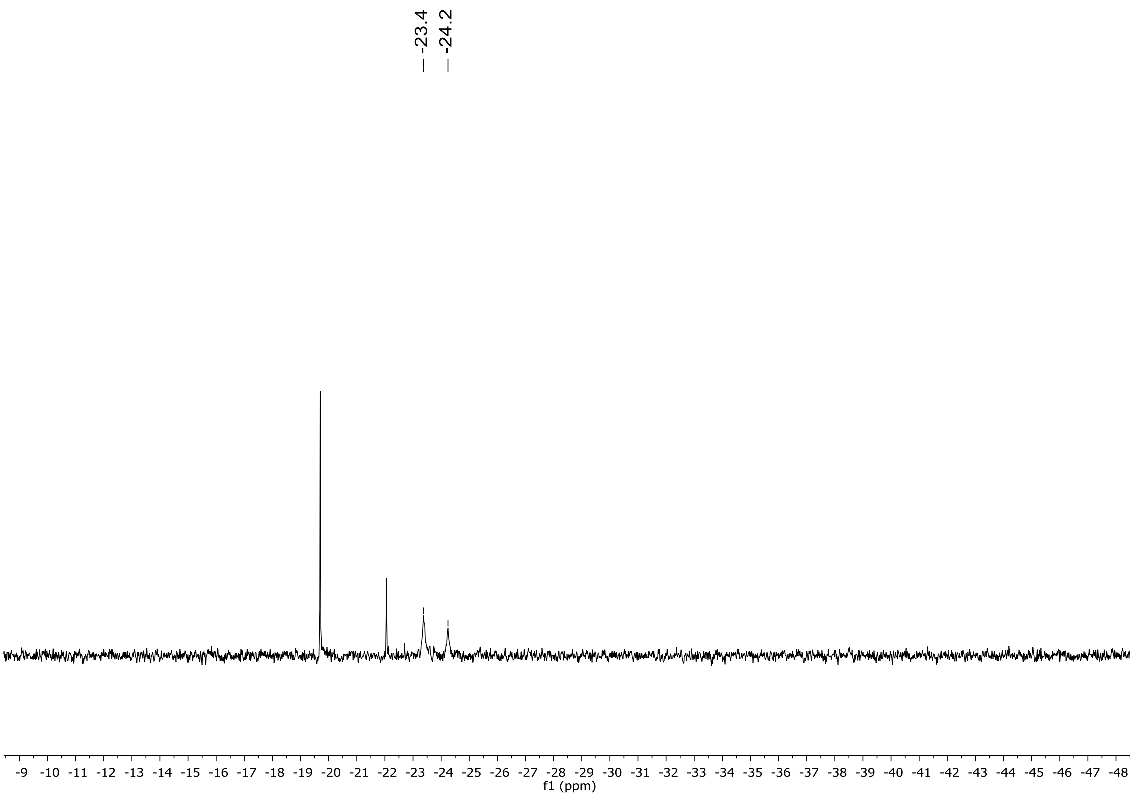


**Figure 10.** $^{29}Si\{^1H\}$ IG NMR spectrum of **[2H][D$_3$OH]** in PhCl after storage at room temperature for several days. Lock with acetone-d$_6$ in a capillary (500 MHz). A reformation of $D_4$ and $D_5$ is observed at $\delta$ = -19.7 and -22.1 ppm.

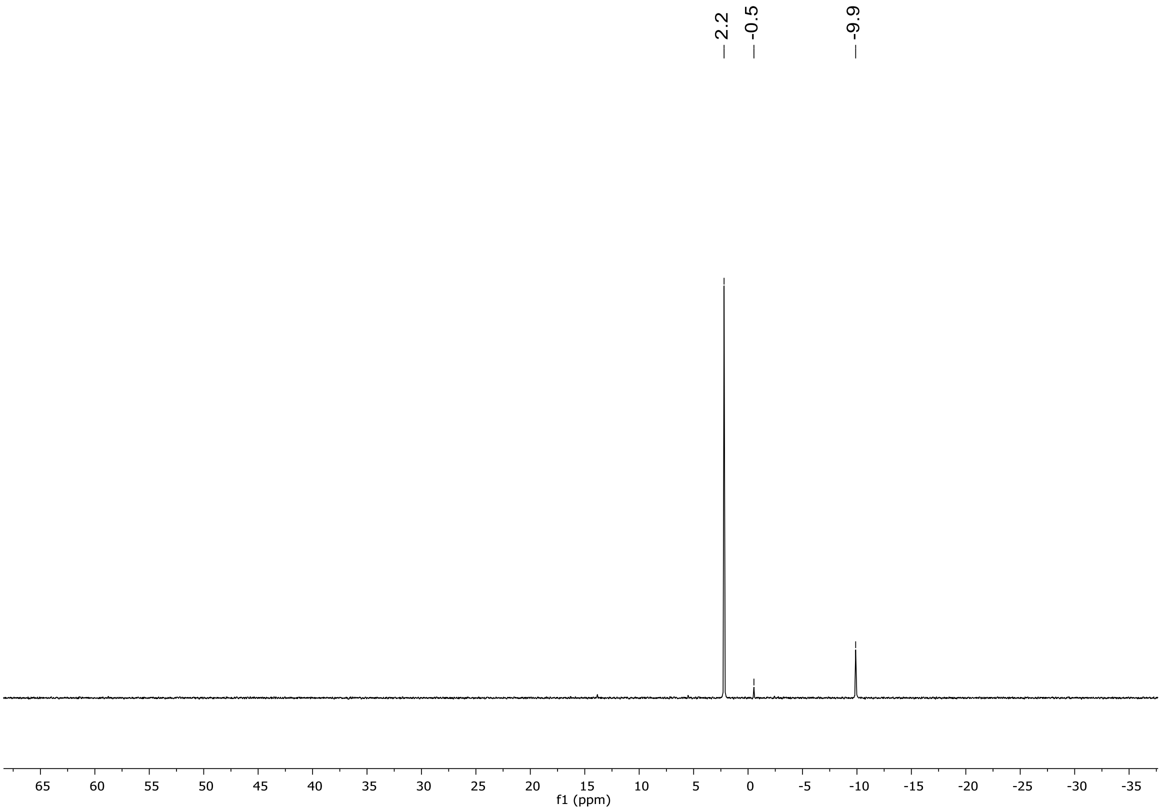


**Figure 11.** $^{31}P$ NMR spectrum of **[2H][D$_3$OH]** in benzene-d$_6$ after storage at room temperature for several days (500 MHz).

### 1.3.3 Synthesis of [$NBu_4$][$D_3OH$]

Octamethylcyclotetrasiloxane ($D_4$, 15.0 mL, 50.6 mmol) is added to solid [$NBu_4$][$OH(OH_2)_{30}$] (16.86 g, 21.1 mmol) and stirred overnight. All volatile compounds are removed in a high vacuum ($10^{-3}$ mbar) over 24 hours and *n*-pentane (25 mL) is added. The suspension is filtered with a frit (P4) and the solid is washed with *n*-pentane (25 mL). The solid is dissolved in diethylether (135 mL) and recrystallized at -28 °C. The product (9.35 g, 19.4 mmol, 92 % based on [$NBu_4$][$OH(OH_2)_{30}$]) is isolated as highly hygroscopic colorless crystals (m.p. 84 °C).

The product decomposes in protic solvents like acetonitrile-$d_3$ and chloroform-$d_1$.

Slow thermolysis in vacuo was observed above 80 °C. The volatile products were collected in a cooling trap at -196 °C and analysed via multinuclear NMR spectroscopy. The cation is deprotonated in a Hofmann elimination with liberation of tributylamine, butene and cyclic siloxanes (mainly $D_4$).

$^1$H NMR ($Et_2O$, rt): $\delta$ [ppm] = 0.1 (s, 12 H, $(H_3C)_2SiOH\text{-}OSi(CH_3)_2$), 0.2 (s, 6 H, $SiO(H_3C)_2SiOSi$), 1.2 (t, $^3J_{HH}$ = 7 Hz, 12 H, $NCH_2CH_2CH_2C\mathbf{H}_3$), 1.7 (t, q, $^3J_{HH}$ = 7 Hz, $^3J_{HH}$ = 7 Hz, 8 H, $NCH_2CH_2C\mathbf{H}_2CH_3$), 2.0 (m, 8 H, $NCH_2C\mathbf{H}_2CH_2CH_3$), 3.8 (m, 8 H, $NC\mathbf{H}_2CH_2CH_2CH_3$), 14.6 (s, br, SiOH).

$^{13}$C{$^1$H} NMR ($Et_2O$, rt): $\delta$ [ppm] = 0.9 (s, $SiO(H_3C)_2SiOSi$), 2.3 (s, $(H_3C)_2SiOH\text{-}OSi(CH_3)_2$), 13.3 (s, $NCH_2CH_2CH_2\mathbf{C}H_3$), 19.9 (s, $NCH_2CH_2\mathbf{C}H_2CH_3$), 25.0 (s, $NCH_2\mathbf{C}H_2CH_2CH_3$), 59.0 (s, $N\mathbf{C}H_2CH_2CH_2CH_3$).

$^{29}$Si{$^1$H} IG NMR ($Et_2O$, rt): $\delta$ [ppm] = -24.9 (s, $SiO(H_3C)_2\mathbf{Si}OSi$), -24.4 (s, $(H_3C)_2\mathbf{Si}OH\text{-}O\mathbf{Si}(CH_3)_2$).

IR (ATR): $\tilde{\nu}$ [$cm^{-1}$] = 2959 (w), 2939 (vw), 2915 (vw), 2872 (vw), 1497 (vw), 1470 (vw), 1379 (vw), 1255 (w), 1244 (m), 1108 (w), 1046 (s), 1020 (s), 873 (w), 853 (w), 793 (vs), 762 (m), 694 (w), 659 (m), 644 (w), 557 (w), 512 (w), 450 (w).

elemental analysis of $C_{22}H_{55}NO_4Si_3$ (M = 481.9 g/mol): calcd.: C 54.83, H 11.50, N 2.91, Si 17.48; found: C 54.52, H 11.49, N 2.88, Si 17.16.

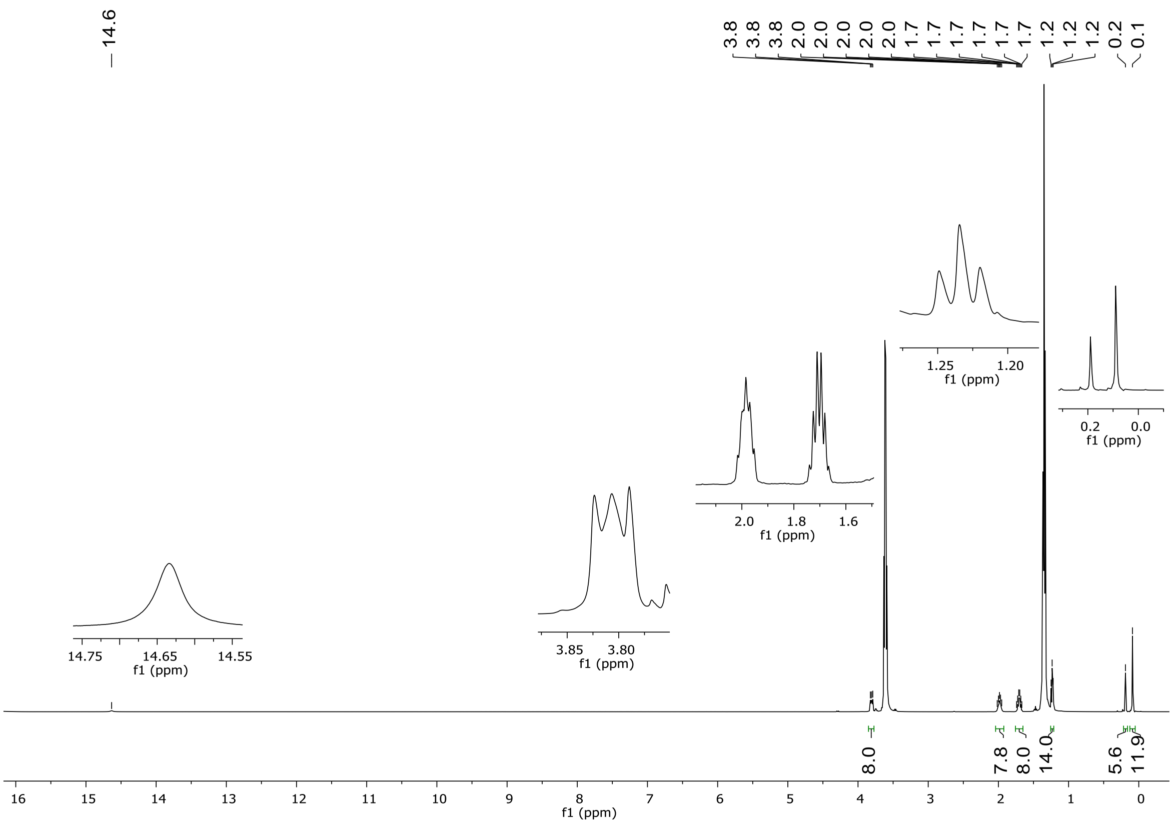

**Figure 12.** $^{1}H$ NMR spectrum of **[NBu$_4$][D$_3$OH]** in $Et_2O$. Lock with acetone-$d_6$ in a capillary (500 MHz).

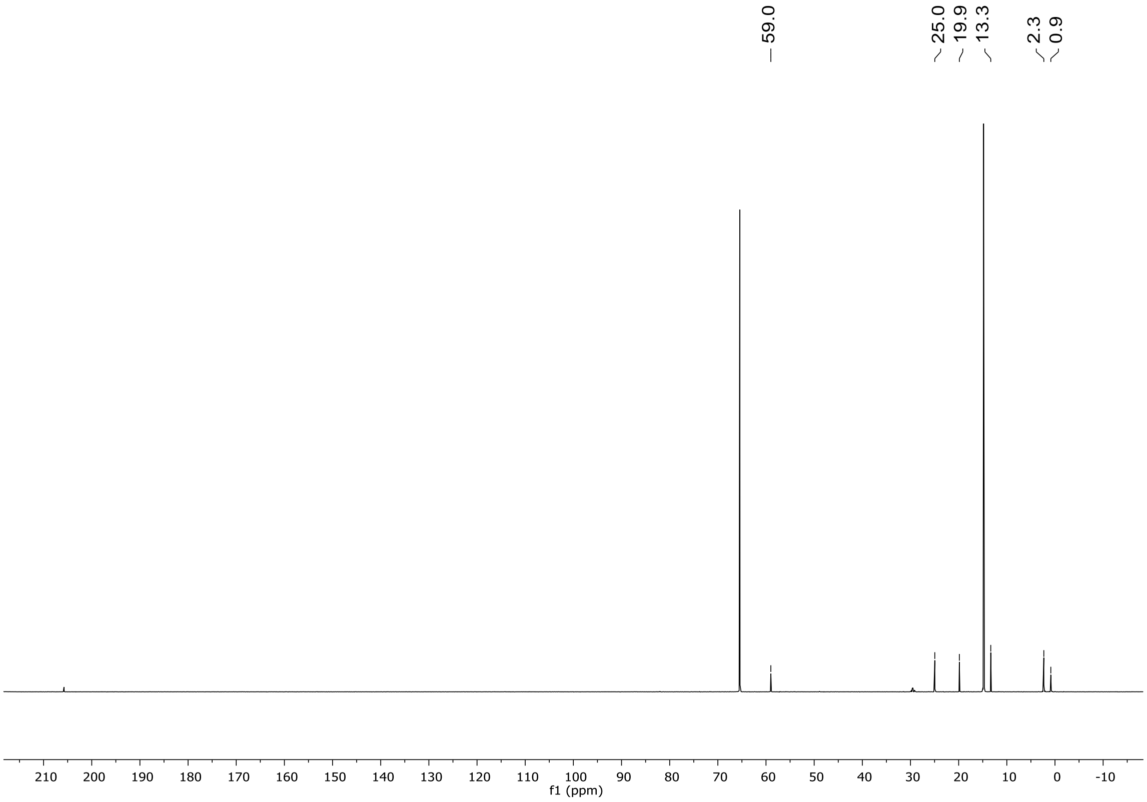

**Figure 13.** $^{13}C\{^{1}H\}$ NMR spectrum of **[NBu$_4$][D$_3$OH]** in $Et_2O$. Lock with acetone-$d_6$ in a capillary (500 MHz).

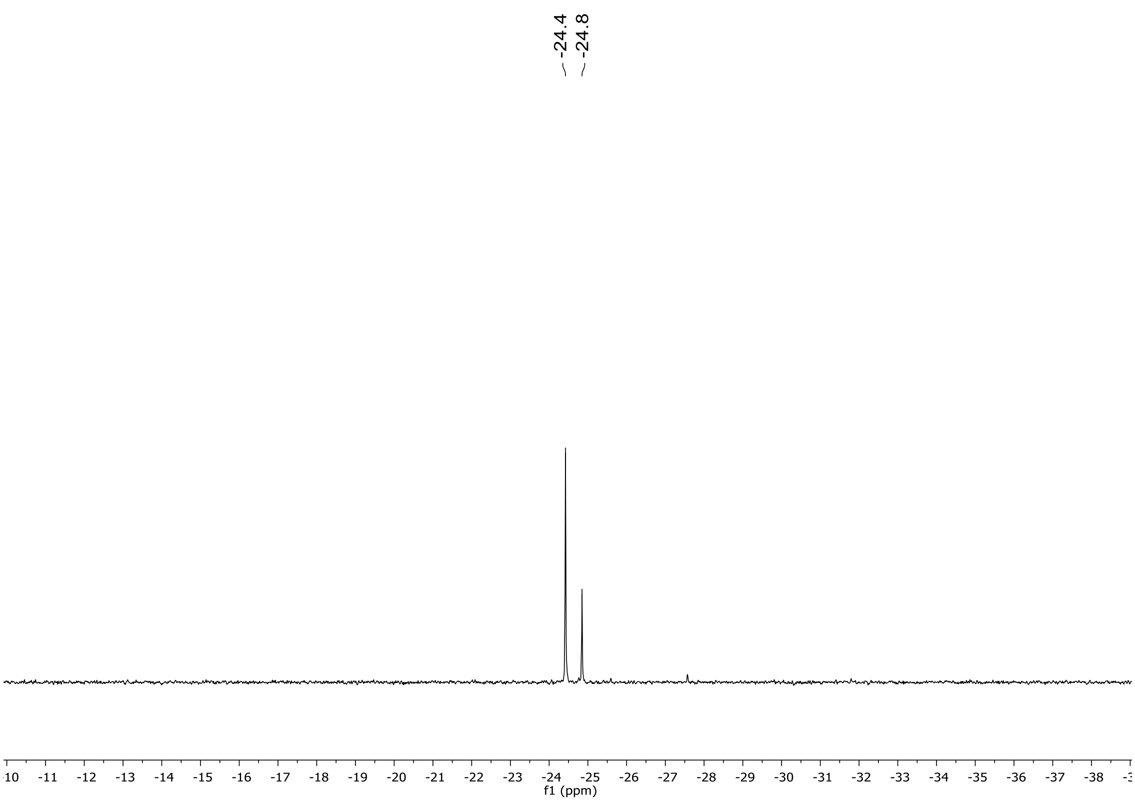


**Figure 14.** $^{29}Si\{^{1}H\}$ IG NMR spectrum of **[NBu$_4$][D$_3$OH]** in $Et_2O$. Lock with acetone-$d_6$ in a capillary (500 MHz).

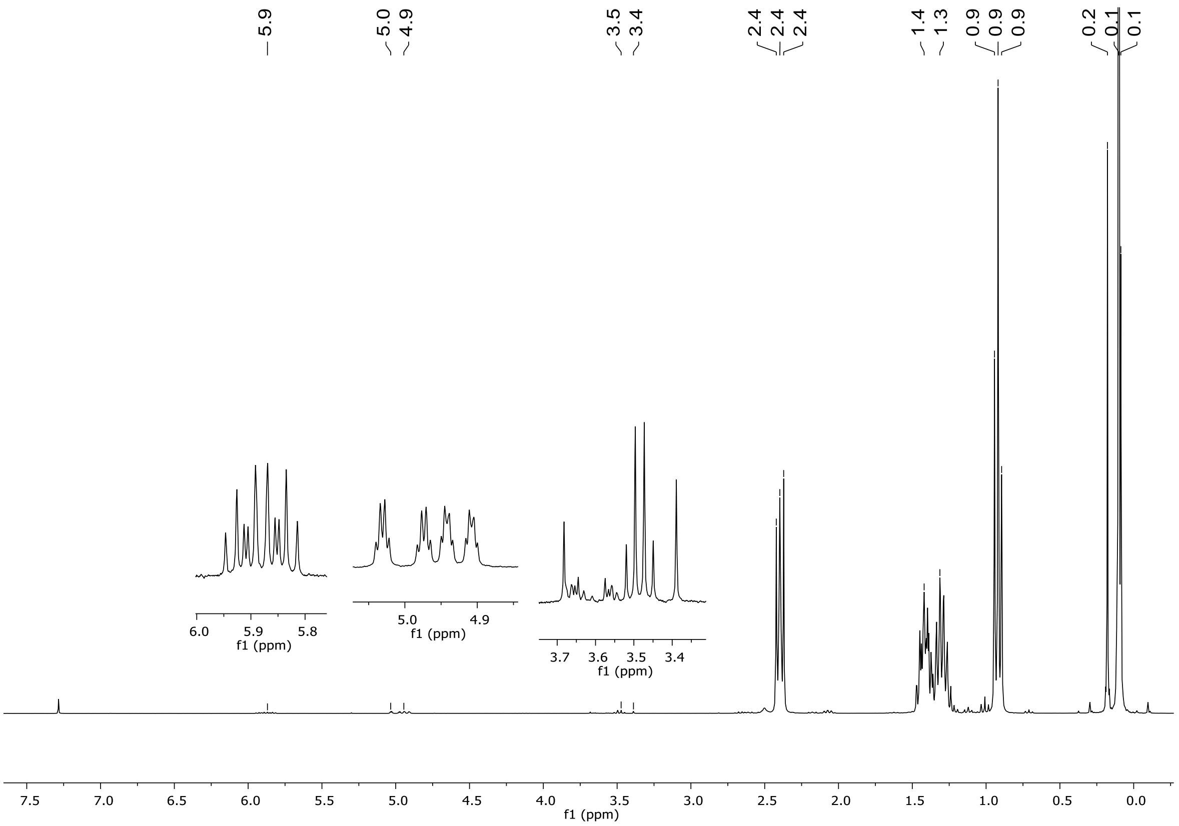


**Figure 15.** $^{1}H$ NMR spectrum of the collected gaseous decomposition products of the vacuum thermolysis of **[NBu$_4$][D$_3$OH]** in $CDCl_3$ (300 MHz).

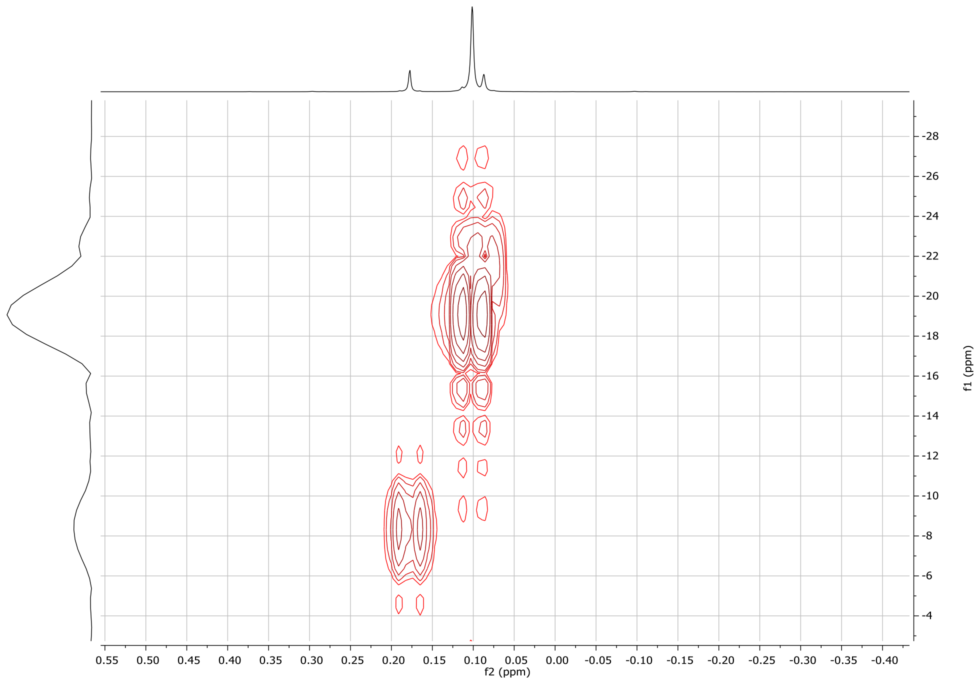


**Figure 16.** $^{1}H^{29}Si$ HMBC NMR spectrum of the collected gaseous decomposition products of the vacuum thermolysis of **[$NBu_4$][$D_3OH$]** in $CDCl_3$ (300 MHz).

### 1.3.4 Synthesis of [$PBu_4$][$D_3OH$]

Octamethylcyclotetrasiloxane ($D_4$, 3.60 g, 12.14 mmol) is added to [$PBu_4$]OH (40 wt.% in $H_2O$, 5.93 g, 8.58 mmol) and stirred for 45 minutes. All volatile compounds are removed in a high vacuum ($10^{-3}$ mbar) over 2 days and the product is recrystallized from $Et_2O$ (20 mL) at -28 °C afterwards. The product (3.13 g, 6.27 mmol, 73 % based on [$PBu_4$]OH) is isolated as highly hygroscopic colorless crystals (m.p. 71 °C).

The product rapidly decomposes by hydrolysis of the tetra-*n*-butylphosphonium cation in ethereal solution and in the solid state.

$^{1}H$ NMR ($Et_2O$, rt): $\delta$ [ppm] = 0.6 (m, 12 H, $(H_3C)_2SiOH\text{-}OSi(CH_3)_2$), 0.7 (s, 6 H, $SiO(H_3C)_2SiOSi$), 1.7 (m, 12 H, $PCH_2CH_2CH_2C\mathbf{H}_3$), 2.3 (m, 16 H, $PCH_2C\mathbf{H}_2C\mathbf{H}_2CH_3$), 3.4 (m, 8 H, $PC\mathbf{H}_2CH_2CH_2CH_3$), 15.7 (s, br, SiOH).

$^{13}C\{^{1}H\}$ NMR ($Et_2O$, rt): $\delta$ [ppm] = -1.3 (m, $SiO(H_3C)_2SiOSi$), 0.0 (m, $(H_3C)_2SiOH\text{-}OSi(CH_3)_2$), 11.0 (s, $PCH_2CH_2CH_2\mathbf{C}H_3$), 16.4 (d, $^{1}J_{PC}$ = 47 Hz, $P\mathbf{C}H_2CH_2CH_2CH_3$), 22.0 (d, $^{2}J_{PC}$ = 15 Hz, $PCH_2\mathbf{C}H_2CH_2CH_3$), 22.2 (d, $^{3}J_{PC}$ = 5 Hz, $PCH_2CH_2\mathbf{C}H_2CH_3$).

$^{29}Si\{^{1}H\}$ IG NMR ($Et_2O$, rt): $\delta$ [ppm] = -26.4 (s, $SiO(H_3C)_2\mathbf{Si}OSi$), -25.5 (s, $(H_3C)_2\mathbf{Si}OH\text{-}O\mathbf{Si}(CH_3)_2$).

$^{31}P$ NMR ($Et_2O$, rt): $\delta$ [ppm] = 33.7 (s).

IR (ATR): $\tilde{\nu}$ [$cm^{-1}$] = 2956 (w), 2901 (vw), 2873 (vw), 1465 (vw), 1423 (vw), 1380 (vw), 1310 (vw), 1247 (m), 1153 (w), 1097 (w), 1045 (s), 1024 (s), 908 (w), 850 (w), 788 (vs), 765 (m), 694 (w), 656 (w), 643 (w), 563 (w), 514 (w), 447 (w).

elemental analysis of $C_{22}H_{55}O_4PSi_3$ (M = 498.9 g/mol): calcd.: C 52.93, H 11.11, P 6.21, Si 16.89; found: C 53.00, H 11.09, P 6.23, Si 16.88.

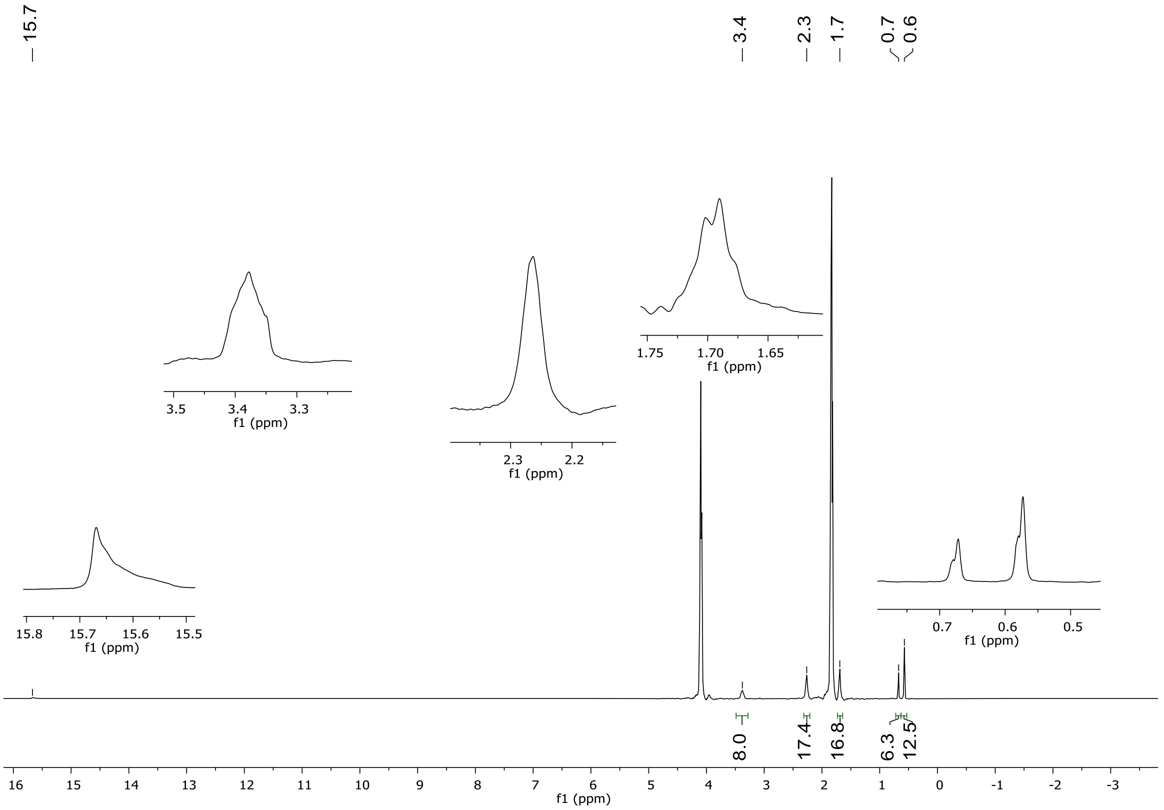


**Figure 17.** $^{1}H$ NMR spectrum of **[PBu$_4$][D$_3$OH]** in $Et_2O$. Lock with acetone-$d_6$ in a capillary (500 MHz).

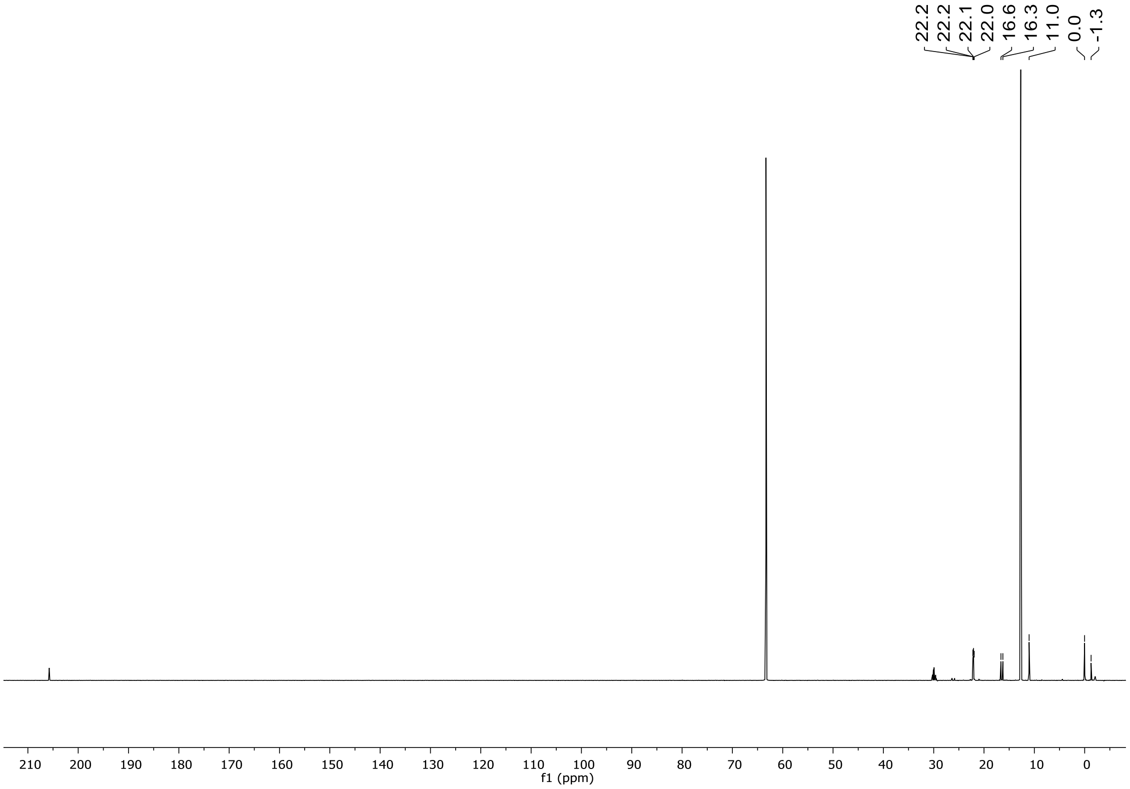


**Figure 18.** $^{13}C\{^{1}H\}$ NMR spectrum of **[PBu$_4$][D$_3$OH]** in $Et_2O$. Lock with acetone-$d_6$ in a capillary (500 MHz).

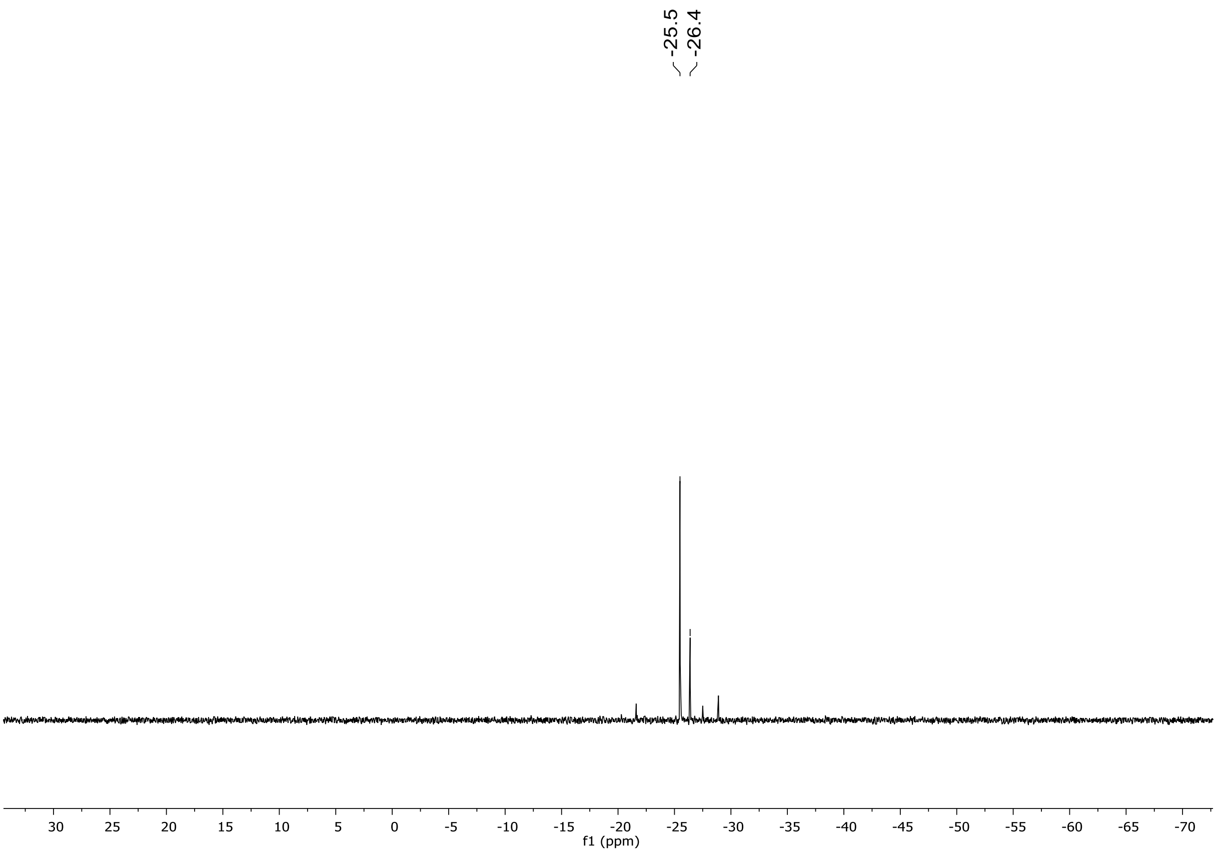


**Figure 19.** $^{29}Si\{^{1}H\}$ IG NMR spectrum of **[PBu$_4$][D$_3$OH]** in $Et_2O$. Lock with acetone-d$_6$ in a capillary (500 MHz).

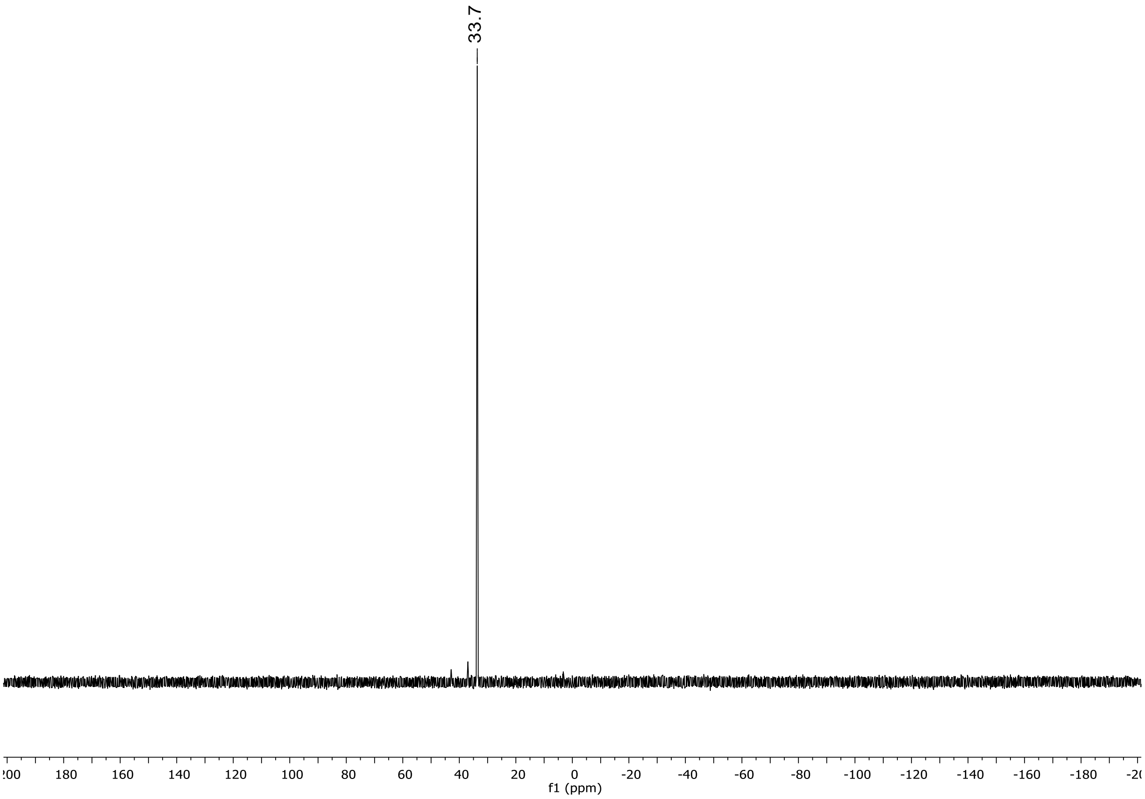


**Figure 20.** $^{31}P$ NMR spectrum of **[PBu$_4$][D$_3$OH]** in $Et_2O$. Lock with acetone-d$_6$ in a capillary (500 MHz).

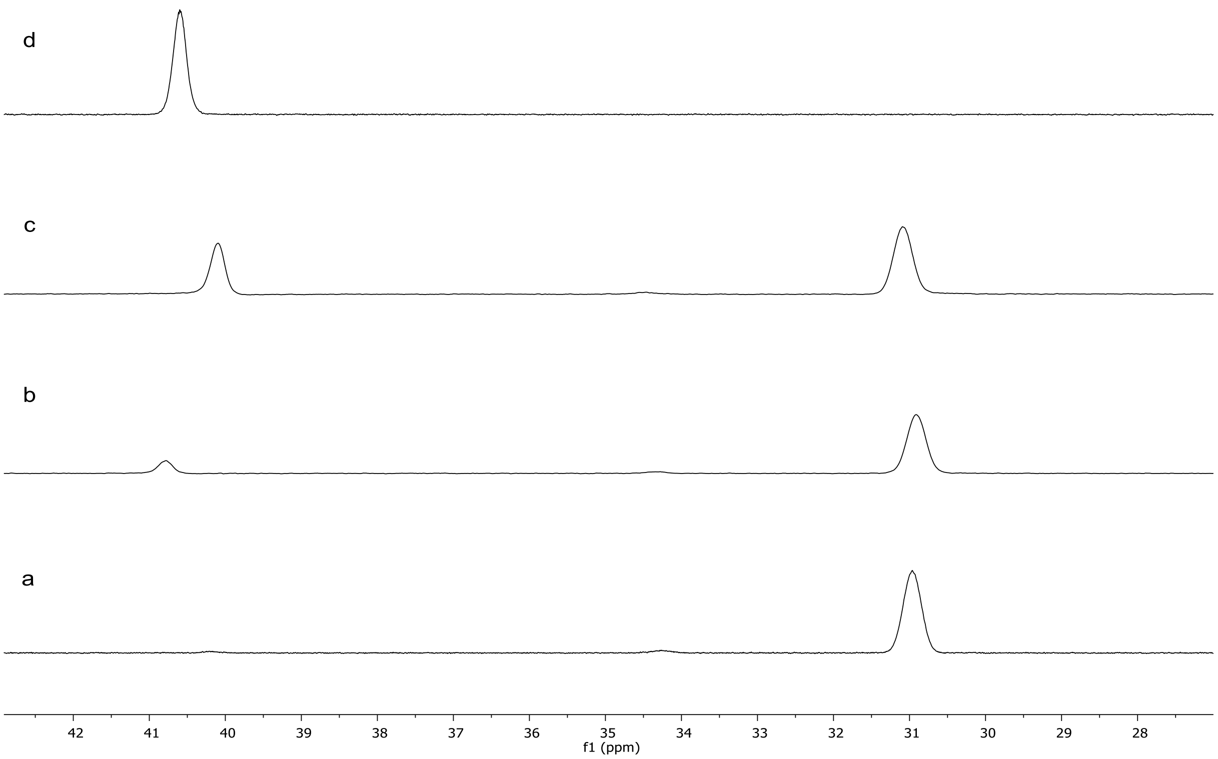


**Figure 21.** $^{31}P$ NMR spectra of the time dependent hydrolysis of **[PBu$_4$][D$_3$OH]** in $Et_2O$. Lock with acetone-$d_6$ in a capillary (500 MHz). $^{31}P$ NMR spectra measured: a) immediately; b) after a few hours; c) over night; d) after several days at ambient temperature.

### 1.3.5 Synthesis of $[NMe_4][D_3OH]_{1/\infty}$

Hexamethylcyclotrisiloxane ($D_3$, 1.38 g, 6.21 mmol) is dissolved in $Et_2O$ (7 mL) and $[NMe_4]OH$ (25 wt.% in MeOH, 2.27 g, 6.23 mmol) is added. The obtained suspension becomes clear while stirring overnight. All volatile compounds are removed in a high vacuum ($10^{-3}$ mbar). The product (1.86 g, 5.93 mmol, 95 % based on $[NMe_4]OH$) is obtained as a colorless, highly hygroscopic sticky oil. Crystallization from ethereal solution at -28 °C affords a highly hygroscopic microcrystalline solid (m.p. 75 °C).
The product rapidly decomposes at ambient temperature under hydrolysis of the tetramethylammonium cation, which is accompanied by a color change to yellow and a strong amine odor.

$^{1}H$ NMR (PhCl, rt): $\delta$ [ppm] = -0.4 to -0.3 (m, 18 H, $(H_3C)_2SiO$), 2.7 (s, 12 H, $N(CH_3)_4$), 13.7 (s, br, SiOH).
$^{13}C\{^{1}H\}$ NMR (PhCl, rt): $\delta$ [ppm] = 1.2 (s, $SiO(H_3C)_2SiOSi$), 2.5 (s, $(H_3C)_2SiOH$-$OSi(CH_3)_2$), 54.3 (s, $N(CH_3)_4$).
$^{29}Si\{^{1}H\}$ IG NMR (PhCl, rt): $\delta$ [ppm] = -28.3 (s), -27.8 (s), -26.2 (s), -26.0 (s), -24.7 (s, $SiO(H_3C)_2$**Si**OSi), -24.1 (s, $(H_3C)_2$**Si**OH-O**Si**$(CH_3)_2$).
IR (ATR): $\tilde{\nu}$ [$cm^{-1}$] = 3021 (vw), 2953 (vw), 2897 (vw), 2819 (vw), 1493 (w), 1409 (vw), 1248 (m), 1014 (s), 948 (m), 920 (m), 901 (m), 845 (m), 766 (vs), 661 (m), 552 (w), 457 (w).

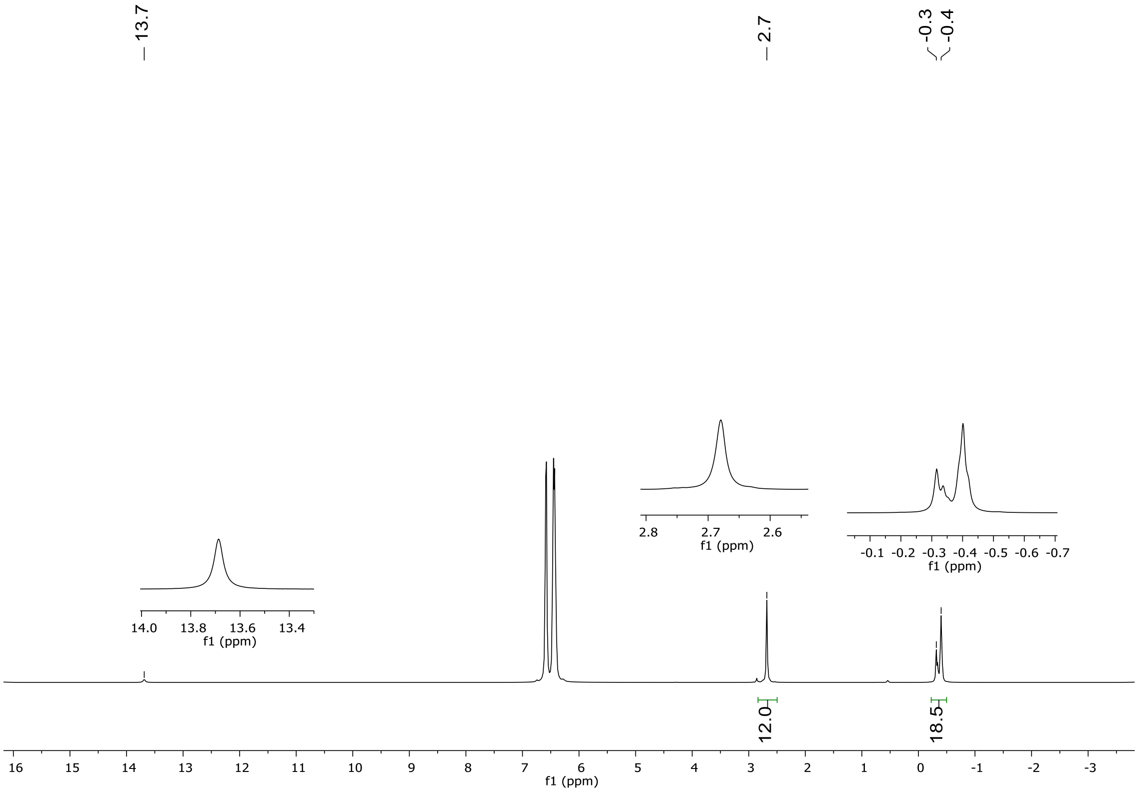


**Figure 22.** $^{1}H$ NMR spectrum of **[NMe$_4$][D$_3$OH]$_{1/\infty}$** in PhCl. Lock with acetone-d$_6$ in a capillary (500 MHz).

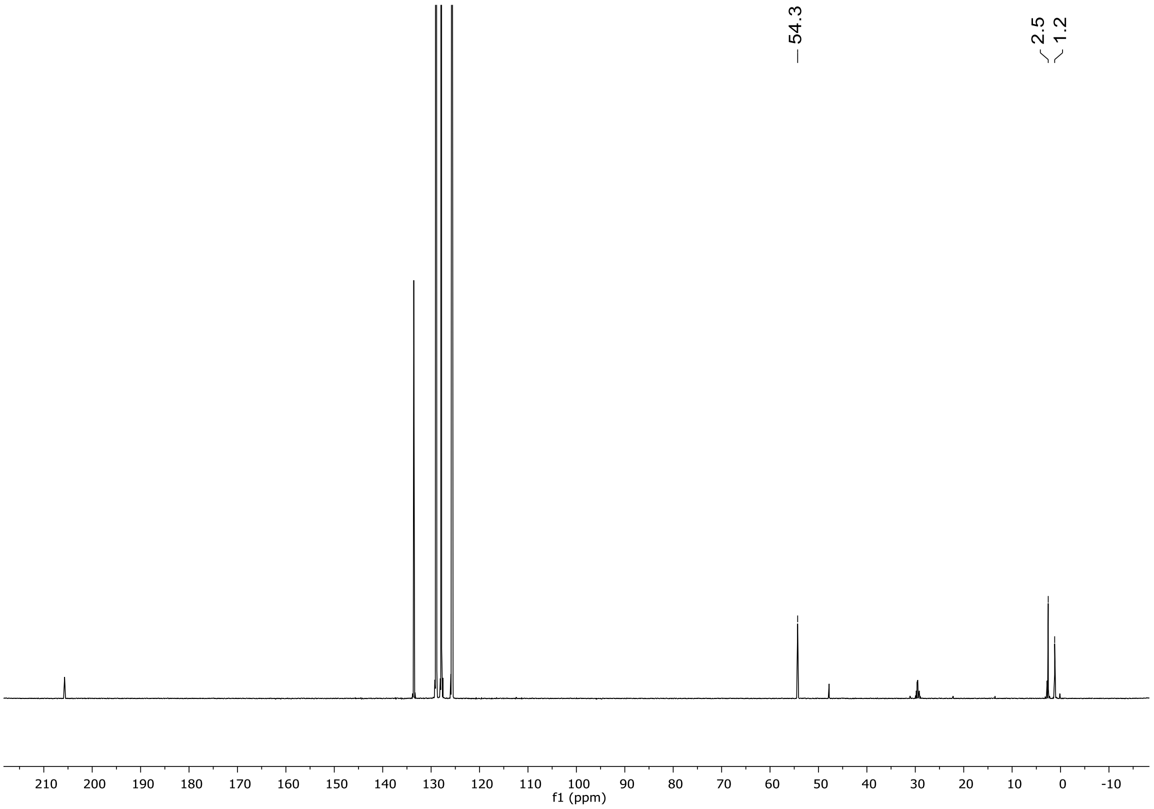


**Figure 23.** $^{13}C\{^{1}H\}$ NMR spectrum of **[NMe$_4$][D$_3$OH]$_{1/\infty}$** in PhCl. Lock with acetone-d$_6$ in a capillary (500 MHz).

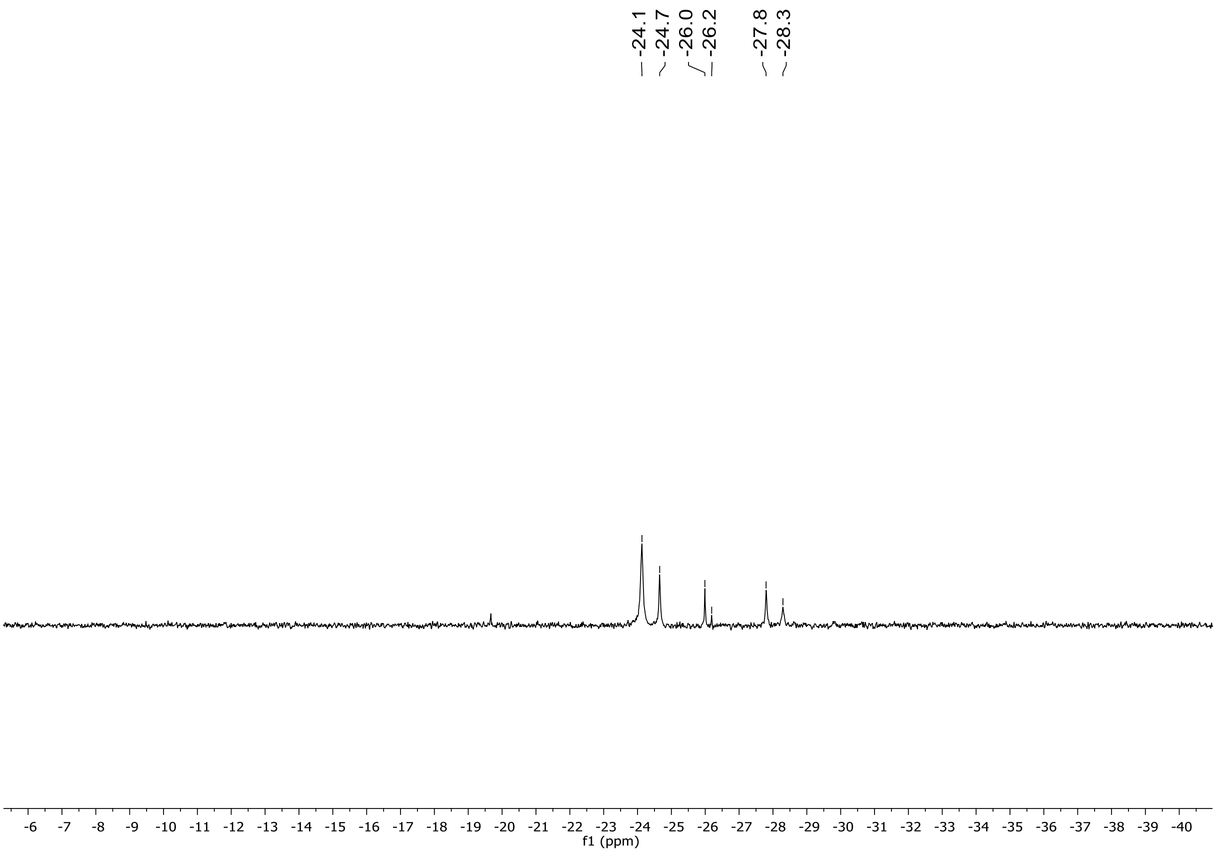


**Figure 24.** $^{29}Si\{^1H\}$ IG NMR spectrum of **[NMe$_4$][D$_3$OH]$_{1/\infty}$** in PhCl. Lock with acetone-d$_6$ in a capillary (500 MHz).

## 1.4 Details on the X-Ray Diffraction

The crystal data were collected on a Rigaku Supernova diffractometer (Cu-$K_\alpha$ radiation ($\lambda$ = 154.184 pm) at 100.0(2) K).

Using Olex2[1], the structures were solved with the ShelXT[2] structure solution program using direct methods and refined with the ShelXL[3] refinement package using least squares minimization. The donor hydrogen atoms were refined isotropically in all these structures.

In **[1H][$D_3$OH]**, N13, C35-C38 are disordered in a ratio of 94:6, the minor occupied atoms were restrained using ISOR. The P4-N13 and P4-N13B distances were restrained to be same as well as the distances O1-HA and O4-HB. The ratio of HA:HB was refined to 506(6):494(6). Using a model without this disorder, the $U_{eq}$ value of this hydrogen atom becomes unreasonably large and the R-values increase slightly.

Details of the X-ray investigation are given in Tables 1-3. CCDC 1952716 and 2024632-2024637 contain the supplementary crystallographic data for this paper. These data can be obtained free of charge via http://www.ccdc.cam.ac.uk/conts/retrieving.html.

**Table 1.** Structure refinement data of **[1H][$D_3$OH]**, **[2H][$D_3$OH]** and **[$NBu_4$][$D_3$OH]**.

| compound | **[1H][$D_3$OH]** | **[2H][$D_3$OH]** | **[$NBu_4$][$D_3$OH]** |
|---|---|---|---|
| empirical formula | $C_{46}H_{119}N_{13}O_4P_4Si_3$ | $C_{25}H_{65}N_{10}O_4PSi_3$ | $C_{22}H_{55}NO_4Si_3$ |
| *a* / pm | 1101.915(13) | 1127.02(2) | 1063.768(8) |
| *b* / pm | 2358.13(3) | 1885.81(3) | 1805.527(12) |
| *c* / pm | 2495.13(3) | 1975.27(3) | 1546.990(10) |
| α / ° | 90 | 90 | 90 |
| β / ° | 93.5860(11) | 105.7492(18) | 98.0714(7) |
| γ / ° | 90 | 90 | 90 |
| *V* / $10^6$ $pm^3$ | 6470.81(13) | 4040.51(12) | 2941.81(4) |
| Z | 4 | 4 | 4 |
| $\rho_{calc}$ / mg·$mm^{-3}$ | 1.157 | 1.126 | 1.088 |
| crystal system | monoclinic | monoclinic | monoclinic |
| space group | $P2_1/c$ | $P2_1/c$ | $P2_1/n$ |
| color shape | colorless needles | colorless fragment | colorless irregular |
| crystal size / $mm^{-3}$ | 0.29 × 0.09 × 0.05 | 0.08 × 0.06 × 0.01 | 0.24 × 0.15 × 0.12 |
| μ / $mm^{-1}$ | 1.985 | 1.785 | 1.674 |
| F(000) | 2480.0 | 1496.0 | 1072.0 |
| 2θ range for data col. / ° | 5.2 to 144.3 | 6.6 to 153.0 | 7.6 to 152.9 |
| index ranges | $-13 \le h \le 13$<br>$-28 \le k \le 28$<br>$-30 \le l \le 30$ | $-14 \le h \le 14$<br>$-21 \le k \le 23$<br>$-24 \le l \le 20$ | $-13 \le h \le 12$<br>$-22 \le k \le 22$<br>$-19 \le l \le 19$ |
| reflections col. | 74329 | 31666 | 55188 |
| independent refl. | 12607 | 8342 | 6140 |
| R(int) | 0.0321 | 0.0375 | 0.0303 |
| data/restraints/ | 12607/122/719 | 8342/725/463 | 6140/0/491 |

| | | | |
|---|---|---|---|
| parameter | | | |
| goodness-of-fit on $F^2$ | 1.025 | 1.044 | 1.037 |
| $R_1$ / $wR_2$ [I>2σ(I)] | 0.0339/0.0834 | 0.0385/0.1028 | 0.0267/0.0714 |
| $R_1$ / $wR_2$ (all data) | 0.0429/0.0884 | 0.0437/0.1072 | 0.0282/0.0726 |
| $\Delta\rho_{max/min}$ / e Å$^{-3}$ | 0.34/-0.37 | 0.31/-0.47 | 0.30/-0.24 |
| CCDC number | 1952716 | 2024632 | 2024633 |

**Table 2.** Structure refinement data of **[PBu$_4$][D$_3$OH]**, **[NMe$_4$][D$_3$OH]$_{1/\infty}$** and **3**.

| compound | **[PBu$_4$][D$_3$OH]** | **[NMe$_4$][D$_3$OH]$_{1/\infty}$** | **3** |
|---|---|---|---|
| empirical formula | $C_{22}H_{55}O_4PSi_3$, $[C_4H_{10}O]_{squeezed}$ | $C_{10}H_{31}NO_4Si_3$ | $C_{17}H_{40}N_9OP$ |
| *a* / pm | 1159.33(4) | 818.959(10) | 1185.894(12) |
| *b* / pm | 1288.56(5) | 866.405(12) | 1519.387(14) |
| *c* / pm | 1293.82(3) | 2586.53(3) | 2610.80(3) |
| α / ° | 94.440(3) | 90 | 90 |
| β / ° | 100.378(3) | 90 | 98.7960(10) |
| γ / ° | 109.237(3) | 90 | 90 |
| $V$ / $10^6$ pm$^3$ | 1775.45(11) | 1835.27(4) | 4648.89(8) |
| Z | 2 | 4 | 8 |
| $\rho_{calc}$ / mg·mm$^{-3}$ | 1.072 | 1.135 | 1.193 |
| crystal system | triclinic | orthorhombic | monoclinic |
| space group | *P*-1 | $P2_12_12_1$ | *Pc* |
| color shape | colorless irregular | colorless plate | colorless needle |
| crystal size / mm$^{-3}$ | 0.35 × 0.26 × 0.24 | 0.29 × 0.16 × 0.04 | 0.36 × 0.04 × 0.02 |
| μ / mm$^{-1}$ | 1.880 | 2.445 | 1.255 |
| F(000) | 636.0 | 688.0 | 1824.0 |

| | | | |
|---|---|---|---|
| 2θ range for data col. / ° | 7.0 to 154.2 | 6.8 to 1532.0 | 5.8 to 153.1 |
| index ranges | $-14 \leq h \leq 14$<br>$-16 \leq k \leq 16$<br>$-16 \leq l \leq 14$ | $-10 \leq h \leq 10$<br>$-10 \leq k \leq 10$<br>$-32 \leq l \leq 32$ | $-14 \leq h \leq 14$<br>$-19 \leq k \leq 18$<br>$-32 \leq l \leq 32$ |
| reflections col. | 32048 | 48329 | 108757 |
| independent refl. | 7373 | 3822 | 19206 |
| R(int) | 0.0452 | 0.0603 | 0.0611 |
| data/restraints/ parameter | 7373/72/285 | 3822/0/287 | 19206/2/1079 |
| goodness-of-fit on $F^2$ | 1.094 | 1.044 | 1.036 |
| $R_1$ / $wR_2$ [I>2σ(I)] | 0.0536/0.1513 | 0.0238/0.0607 | 0.0387/0.0945 |
| $R_1$ / $wR_2$ (all data) | 0.0588/0.1573 | 0.0254/0.0620 | 0.0473/0.0995 |
| $\Delta\rho_{max/min}$ / e Å$^{-3}$ | 1.08/-0.56 | 0.27/-0.18 | 0.40/-0.27 |
| Flack parameter | - | -0.011(10) | 0.10(2) |
| CCDC number | 2024634 | 2024635 | 2024636 |

**Table 3.** Structure refinement data of **[$NMe_4$][K($D_3OH$)$_2$]**.

| compound | **[$NMe_4$][K($D_3OH$)$_2$]** |
|---|---|
| empirical formula | $C_{32}H_{100}K_2N_2O_{16}Si_{12}$ |
| *a* / pm | 1178.68(5) |
| *b* / pm | 1216.96(6) |
| *c* / pm | 1216.96(6) |
| α / ° | 100.829(4) |
| β / ° | 93.251(3) |
| γ / ° | 118.521(5) |

| | |
|---|---|
| $V$ / $10^6$ pm$^3$ | 1649.99(14) |
| Z | 1 |
| $\rho_{calc}$ / mg·mm$^{-3}$ | 1.192 |
| crystal system | triclinic |
| space group | *P*-1 |
| color shape | colorless irregular |
| crystal size / mm$^{-3}$ | 0.21 x 0.11 x 0.08 |
| μ / mm$^{-1}$ | 3.793 |
| F(000) | 640 |
| 2θ range for data col. / ° | 6.75 to 153.40 |
| index ranges | $-14 \leq h \leq 14$<br>$-15 \leq k \leq 15$<br>$-17 \leq l \leq 17$ |
| reflections col. | 25456 |
| independent refl. | 6794 |
| R(int) | 0.0759 |
| data/restraints/ parameter | 6794/0/489 |
| goodness-of-fit on $F^2$ | 1.022 |
| $R_1$ / $wR_2$ [I>2σ(I)] | 0.0409/0.0869 |
| $R_1$ / $wR_2$ (all data) | 0.0644/0.0974 |
| $\Delta\rho_{max/min}$ / e Å$^{-3}$ | 0.54/-0.07 |
| Flack parameter | - |
| CCDC number | 2024637 |

## References

[1] O. V. Dolomanov, L. J. Bourhis, R. J. Gildea, J. A. K. Howard, H. Puschmann, *J. Appl. Cryst.* **2009**, *42*, 339.

[2] G. M. Sheldrick, *Acta Cryst. A* **2015**, *71*, 3.

[3] G. M. Sheldrick, *Acta Cryst. C* **2015**, *71*, 3.
